# HAMP Domain Conformers That Propagate Opposite Signals in Bacterial Chemoreceptors

**DOI:** 10.1371/journal.pbio.1001479

**Published:** 2013-02-12

**Authors:** Michael V. Airola, Nattakan Sukomon, Dipanjan Samanta, Peter P. Borbat, Jack H. Freed, Kylie J. Watts, Brian R. Crane

**Affiliations:** 1 Department of Chemistry and Chemical Biology, Cornell University, Ithaca, New York, United States of America; 2 Center for Advanced ESR Studies, Cornell University, Ithaca, New York, United States of America; 3 Division of Microbiology and Molecular Genetics, Loma Linda University, Loma Linda, California, United States of America; Princeton University, United States of America

## Abstract

HAMP domains are signal relay modules in >26,000 receptors of bacteria, eukaryotes, and archaea that mediate processes involved in chemotaxis, pathogenesis, and biofilm formation. We identify two HAMP conformations distinguished by a four- to two-helix packing transition at the C-termini that send opposing signals in bacterial chemoreceptors. Crystal structures of signal-locked mutants establish the observed structure-to-function relationships. Pulsed dipolar electron spin resonance spectroscopy of spin-labeled soluble receptors active in cells verify that the crystallographically defined HAMP conformers are maintained in the receptors and influence the structure and activity of downstream domains accordingly. Mutation of HR2, a key residue for setting the HAMP conformation and generating an inhibitory signal, shifts HAMP structure and receptor output to an activating state. Another HR2 variant displays an inverted response with respect to ligand and demonstrates the fine energetic balance between “on” and “off” conformers. A DExG motif found in membrane proximal HAMP domains is shown to be critical for responses to extracellular ligand. Our findings directly correlate in vivo signaling with HAMP structure, stability, and dynamics to establish a comprehensive model for HAMP-mediated signal relay that consolidates existing views on how conformational signals propagate in receptors. Moreover, we have developed a rational means to manipulate HAMP structure and function that may prove useful in the engineering of bacterial taxis responses.

## Abstract 2

How do cell-surface receptors transmit signals into cells? This study resolves how signal relay occurs through the HAMP domains of bacterial chemoreceptors by causing them to switch between two conformational states.

## Author Summary

A central question in biological signal transduction is how cell-surface receptors transmit signals from the outside world across cell membranes and into the cells themselves. In bacteria and lower eukaryotes such receptors are composed of individual modules responsible for specific functions (e.g., sensing, relay, or output). HAMP domains act as the signal relay modules in many receptors, physically bridging input and output components and transferring signals between them. Through a combination of crystallographic, biophysical, spectroscopic, and functional studies we are able to associate two structurally defined HAMP conformational states with functional “on” and “off” signals in bacterial chemoreceptors, and thereby resolve the mechanism by which HAMPs can relay information. The two states differ in both their structure and dynamics and appear to enforce their properties on downstream output modules. Chemoreceptors allow bacteria to track chemical gradients with exquisite sensitivity and dynamic range; we further show that the response to chemoattractant depends critically on specific HAMP residues close to the membrane. Finally, based on the switching mechanism, we design and generate an inverse signaling HAMP domain that provides a new tool to engineer bacterial responses and may be especially advantageous in remediation efforts for directing bacteria towards chemicals that are normally repellants.

## Introduction

The ability of single-celled organisms to sense, respond to, and adapt to their changing environment requires receptor proteins to convert extracellular signals into cellular responses [1]. Central to many of these signal transduction systems are HAMP domains, which act to couple sensory and output domains in over 26,000 different receptor proteins [2]. In transmembrane receptors, HAMP domains connect to transmembrane helices entering the cytoplasm and translate chemical, photo, and thermo stimuli to the output of cytoplasmic catalytic domains (mainly histidine kinases, adenylyl cyclases, methyl-accepting chemotaxis proteins [MCPs], and phosphatases) [3]. Deletion of HAMP domains disrupts the link between input and output units, generating receptors incapable of switching activity states upon stimulation [4].

HAMP domains are small modules, approximately 50 amino acids, that dimerize to form an entirely parallel four-helix bundle with two helices (AS1 and AS2) supplied from each subunit [3]. The AS1 and AS2 helices form a seven-residue pattern characteristic of coiled coils, termed a heptad repeat, with the repeat residues labeled *a* through *g*, and with the *a* and *d* positions hydrophobic in nature and pointing inward to form a buried core [5]. A semi-structured connector separates the AS1 and AS2 helices and contains two conserved hydrophobic residues, termed HR1 and HR2 [6]. A spectrum of HAMP domain structures and conformations is now characterized for native and mutant HAMP domains, the most divergent of which differ by helix rotation, helix translation, and helix–helix crossing angle [5],[7]–[11]. Importantly, the transmembrane helices of characterized HAMP-containing receptors are known to undergo small amplitude translations or rotations during signal transduction [12],[13].

**Figure 1:**
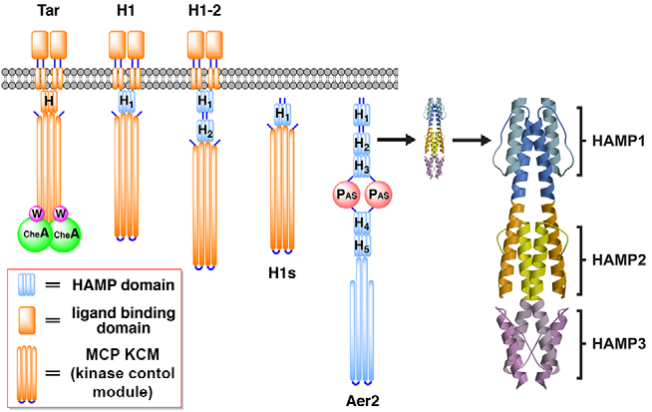
Schematic of Aer2-Tar Chimeras. The HAMP domain of Tar was replaced with single and poly-HAMP domains from Aer2 to generate chimeric receptors. Transmembrane ATCs (e.g., H1) contained the ligand binding domain and Tar KCM, both of which are necessary for modulating CheA kinase activity in response to aspartate. Soluble ATCs (e.g., H1s) comprised fusions of the Aer2 HAMP domains with only the Tar KCM. The structure of the three-unit Aer2 poly-HAMP domain (Protein Data Bank code 3LNR) is shown on the right, with HAMP1 (blue), HAMP2 (yellow/orange), and HAMP3 (purple) colored accordingly.

The function and mechanism of HAMP domains have been most intensively studied in MCPs, which regulate bacterial chemotaxis and are archetypal models of bacterial transmembrane signaling [3]. Overall, MCPs have a modular construction comprising an extracellular ligand-sensing domain, a transmembrane helical region, a membrane proximal HAMP or tandem HAMP domain, and a kinase control module (KCM) containing the adaptation region and kinase coupling tip (Figure 1) [14]. MCPs sense chemical gradients to direct bacterial cells towards or away from attractants and repellents through allosteric activation and inhibition of the histidine kinase CheA. CheA phosphorylates the response regulator CheY to generate CheY-P. Depending on the ratio of CheY to CheY-P, flagella rotate counterclockwise (CCW) or clockwise (CW). Cells bias their movement by alternating between bursts of straight swimming (CCW rotation) and tumbling (CW rotation) [3].

MCP activity is also modulated by an adaptation system composed of the methyltransferase CheR and the methylesterase CheB. CheR and CheB respectively methylate and demethylate specific Glu residues to compensate for ligand binding and to reverse signals to the kinase relayed by the HAMP domain [4]. The predominant *Escherichia coli* chemoreceptors Tar and Tsr have four (or five) Glu methylation sites on each subunit (EEEE); however, two sites are expressed as Gln residues (QEQE) and are subsequently deamidated by CheB [15]. By reestablishing an optimum response set point, the adaptation system allows MCPs to sense a wide concentration range of stimulants with remarkable sensitivity [14]. Importantly, the adaptation system compensates for perturbations to receptor activity, i.e., demethylation/deamidation will thus attempt to “turn down” kinase-on states and methylation will “turn up” kinase-off states. Thus, only in the absence of the adaptation system (CheRB− cells) can the unbiased activity state of a given receptor be established [16].

**Figure 2:**
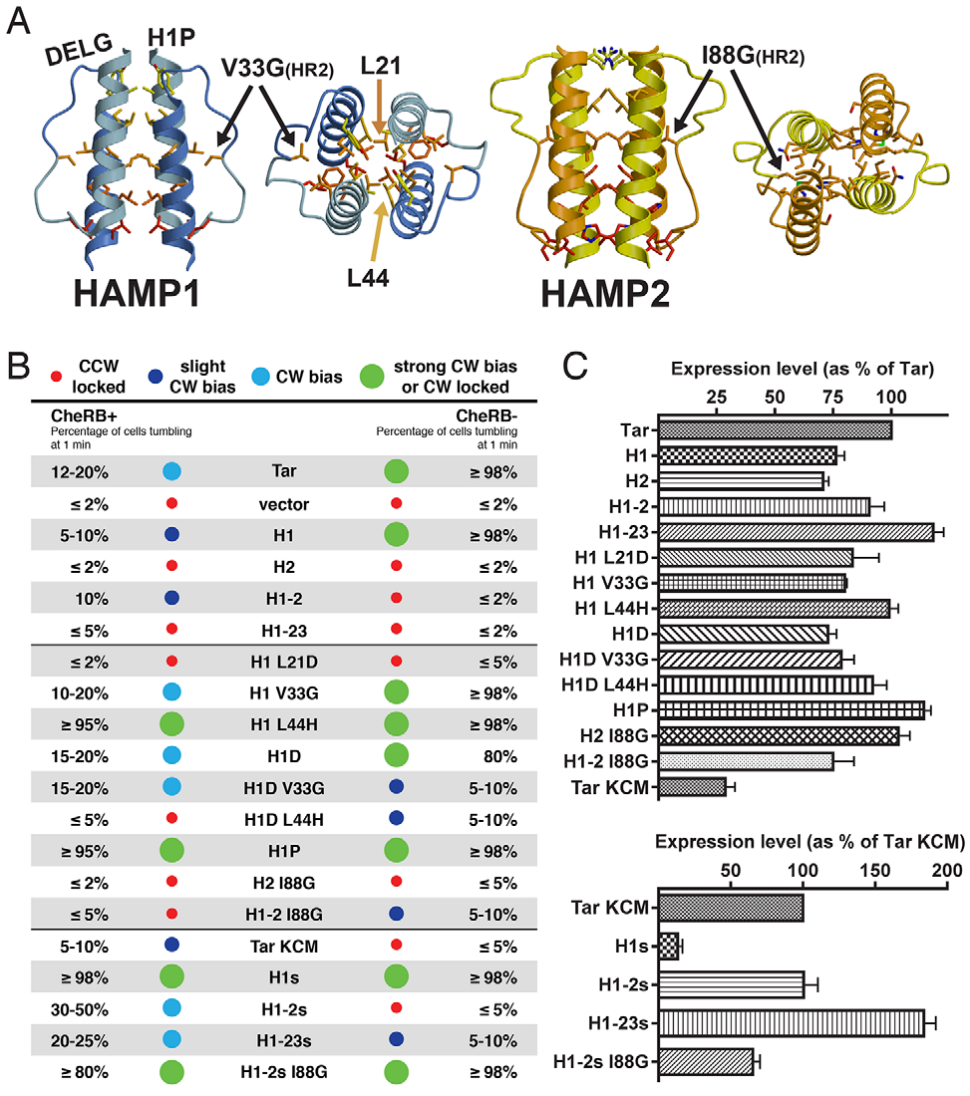
Signaling biases and expression levels of ATC receptors. (A) Structures of HAMP1 and HAMP2, highlighting positions of mutations reported in this study. HR2 (I88G) plays a prominent role in the HAMP2 hydrophobic core, inserting into the HAMP bundle between AS1 and AS2, while HR2 (V33G) in HAMP1 appears dispensable for bundle stability as it resides on the domain periphery. L21 and L44 occupy core heptad positions inside the HAMP bundle. Membrane-associated HAMP domains contain a highly conserved DExG motif at the connector-AS2 junction and a less conserved Pro residue between TM2 and AS1. (B) Tumbling biases of transmembrane and soluble ATC receptors quantified by temporal assays in CheRB+ and CheRB− cells. Signaling biases are grouped into four categories: (1) CCW locked (<5% CW), (2) slight CW bias (5%–10% CW), (3) CW bias (10%–50% CW), and (4) strong CW bias (50%–95% CW) or CW locked (>95% CW). Temporal assays confirm H1 and H1-2 induce opposite outputs. The L44H mutation generates a CW locked receptor with or without the adaptation system. The soluble receptors H1s and H1-2s generate more distinct CW and CCW locked phenotypes in CheRB− cells than their transmembrane counterparts. Mutation of HR2 in H1-2s I88G switches receptor signaling from CCW to CW locked, which is consistent with HR2 stabilizing the CCW HAMP2 conformer. (C) Expression levels of ATC receptors in CheRB+ (BT3388) cells, normalized to that of Tar for transmembrane receptors and that of Tar KCM for soluble receptors.

The first HAMP domain structure, Af1503, was determined by nuclear magnetic resonance (NMR) from an orphan receptor from *Archaeoglobus fulgidus* [5]. We subsequently determined the structure of a poly-HAMP domain composed of three concatenated HAMP units from the *Pseudomonas aeruginosa* soluble receptor Aer2 [7]. The Aer2 HAMP domains are representative of a recently identified sequence cluster that comprises repeating units to form extended, linear poly-HAMP chains [7],[17]. These atypical HAMPs share similar residue conservation and overall structure with membrane-associated HAMP domains but differ in that they lack obvious signal input motifs [17],[18]. Aer2 is a soluble receptor that contains three N-terminal HAMP domains, a gas-sensing, heme-containing PAS domain, two additional HAMP domains, and an MCP KCM (Figure 1) [19]. The three N-terminal HAMP domains of Aer2 (named HAMP1, HAMP2, and HAMP3 from N- to C-terminus) provide examples of two distinctly different conformations: HAMP1 and HAMP3 are similar to the Af1503 NMR structure, whereas HAMP2 has a comparatively distorted four-helix bundle structure in which the AS2 helices approximate a two-helix coiled coil and the AS1 helices splay outward at the C-terminal end. Importantly, a functionally critical hydrophobic residue in the helical connector [6], termed HR2, plays a clear role in stabilizing the HAMP2 structure by inserting between the AS1/AS2 helices, but remains on the periphery and appears dispensable in the HAMP1-like conformers (Figure 2). The alternating and divergent conformations of the Aer2 HAMP moieties led us to hypothesize that HAMP1 and HAMP2 may represent two sides of a conformational switch that could send opposing signals to an output domain [7].

Several additional mechanisms have been proposed for HAMP domain signal relay. Functional characterization of an extensive library of HAMP mutants in the *E. coli* serine receptor Tsr has led to a model of HAMP function in which activity states of HAMP variants lie on a biphasic curve of domain stability [16],[20]. Variants predicted to be very unstable or very stable do not activate CheA (CCW flagellar rotation), whereas variants of intermediate stability activate CheA (CW flagellar rotation). The “stable” CCW(A) state is proposed to be the functional off state, and the metastable CW state the physiological on state. The very unstable CCW(B) state arises from drastic mutations that perturb HAMP properties out of its natural range. The biphasic model explains several unusual variants in which methylation and demethylation have inverted effects on the ability to activate CheA. Correlation of the residue substitutions with domain stability is largely inferred based on the effects the mutations are likely to have on known HAMP structures, particularly that of Af1503 [16].

Our goal is to assign the conformational properties of the HAMP states in bacterial chemotaxis receptors that produce CW and CCW rotational behavior. Corresponding experiments have been carried out with the Af1503 HAMP grafted into chimeras of adenylate cyclase and sensor kinase output domains [8],[9],[21],[22]. There, crystallographic and NMR spectroscopy data on isolated HAMP mutants were correlated with their ability to modulate cyclase or kinase activity. These data in part supported a model in which helical rotation within HAMP is responsible for downstream signaling; however, the conformational differences found among the crystallized HAMP mutants were more complex than simple helix rotations, and the correlation between the amount of rotation at the C-terminus of AS2 and the activity of the receptor was not striking across the entire set of variants tested.

A prime problem in structure–function studies of HAMP domains is the difficulty in mapping structural and biophysical properties of isolated HAMP domains to their functional states in transmembrane receptors. The problem is compounded by the sensitivity of HAMP domains to perturbations and the possibility that different conformational states produce similar outputs. The question then becomes: what are the essential conformational features HAMP domains enforce on output domains to set their activity states?

Here we investigate the downstream signaling and functional capabilities of structurally defined Aer2 HAMP domains in chimera MCP transmembrane receptors. We report that the two structural HAMP domain variants, HAMP1 and HAMP2, give rise to opposite CW and CCW downstream signals in vivo, and using spin-labeling distance measurements, we find that HAMP domains assume both conformations in solution. Crystal structures of HAMP domain mutants locked in activating signaling states confirm the structural relationship and provide insight into mechanisms of disrupting mutagenesis. Mutation of HR2, which is selectively important for the HAMP2 (CCW) conformation, shifts receptor bias towards a CW state. In addition, a reconstituted, functional HAMP1 receptor confirms the role of the DExG signal input motif [17]. We also identify a novel inverse signaling HAMP1 mutant receptor with the same degree of ligand sensitivity as endogenous MCPs. Our collective results support a model in which HAMP domains switch primarily between the two conformations to propagate signals in bacterial chemoreceptors.

## Results

### Two Structurally Characterized HAMP Domain Conformers Produce Opposite Downstream Signals

To understand HAMP signaling states, the preferred course would be to correlate the extensive genetic and functional data for the HAMP domains of the *E. coli* chemoreceptors Tar and Tsr with their structural and biophysical properties. Unfortunately, the *E. coli* HAMPs cannot be produced or studied in isolation or as soluble domain fusions. In contrast, the N-terminal Aer2 HAMP domains are highly amenable to structural characterization, but their contribution to Aer2 signaling is not well defined, and in fact, the function of Aer2 itself is not fully understood [19]. Thus, we have developed a chimeric system in which direct measurements of Aer2 HAMP conformation can be coupled to biological readouts.

Aer2–Tar chimeras (ATCs) were generated by replacing the HAMP domain of the *E. coli* aspartate receptor Tar with single or poly-HAMP domains from Aer2 (Figure 1). These chimeric proteins were then expressed in *E. coli* cells lacking endogenous MCPs, and receptor function was assessed (Figure S1; Table S1). Direct measurements of cell tumbling frequencies (tumbling = CW, smooth swimming = CCW) were employed to confirm the flagellar output states of select receptors (Figure 2). Receptors were scored in terms of percent CW bias, by counting the number of tumbling or smooth swimming cells after 1 min of observation, and were grouped into four categories: (1) CCW locked (<5% CW), (2) slight CW bias (5%–10% CW), (3) CW bias (10%–50% CW), and (4) strong CW bias (50%–95%) or CW locked (>95% CW). Prior to observation, cells were allowed to adapt for 5 min. Two strains that either contained (CheRB+, BT3388) or were devoid of (CheRB−, UU2610) the methylation system were used to harbor ATC receptors. Changes in behavior between CheRB+ and CheRB− indicate that the receptors assemble into functional clusters capable of activating CheA and responding to the adaptation system, at least to some degree. The CheRB− background provides an indication of intrinsic receptor activity in the absence of receptor modification. Select ATC receptors tested in strain UU2612, which is CheRB+ but otherwise isogenic to UU2610, gave nearly identical responses to those expressed in BT3388.

As expected, vector controls in both CheRB+ and CheRB− cells were exclusively smooth swimming (≤2% CW) (Figure 2). Tar produced a modest CW bias in CheRB+ cells, and a CW locked phenotype in CheRB− cells. Thus, Tar alone is strongly CheA activating in its unmodified form (QEQE), whereas the adaptation system deactivates this receptor, largely by deamidating two of the methylation sites (QEQE to EEEE). This is similar to expression of Tsr, which produces a CW bias phenotype (25% CW) in CheRB+ cells and a strong CW bias phenotype (75% CW) in CheRB− cells [16].

The H1 receptor, containing HAMP1 in place of the native Tar HAMP, behaved similar to Tar, being slightly CW biased in CheRB+ cells and CW locked in CheRB− cells. On the other hand, the H1-2 receptor, containing HAMP1 and HAMP2 in tandem (the dash in H1-2 denotes the short helical linker) was similar to H1 in CheRB+ cells but CCW locked in CheRB− cells. The contrasting behavior of H1 and H1-2 implies that the two different conformations of HAMP1 and HAMP2 send opposite signals to Tar KCM and elicit different responses from the adaptation system. The remaining unmutated ATCs were nearly exclusively CCW locked in both CheRB+ and CheRB− cells. Although all ATC receptors tested were expressed at normal levels (Figure 2C), inactivity could indicate that these receptors do not assemble into functional clusters and/or are incapable of productive interactions with CheA and CheW. These additional factors may explain why the H1-23 receptor, which would be predicted to share the same HAMP conformer type and output as H1, displayed a CCW locked phenotype. Consequently, we limited our remaining studies to the functional receptors H1 and H1-2.

### Single Residue Substitutions Dramatically Affect ATC Phenotypes

Using our ATC system, we sought to better understand the principles underlying HAMP domain signal transduction by directly comparing in vivo signaling biases with the in vitro physical properties of point mutants that alter domain output. We thus generated single residue substitutions of ATC receptors with consideration of the extensive HAMP mutational data for the Tsr chemoreceptor as a guide. We focused mainly on H1 because of its functionality and the fact that HAMP1 is decoupled from HAMP2/3 in Aer2 1–172 by a short helical linker and hence is less likely to be dependent on HAMP2/3 for stability.

Cellular flagellar responses to single residue substitutions in H1 were varied, with roughly half of the substitutions having effects on signaling bias similar to those seen with equivalent substitutions in Tsr, and half having opposite effects (Table S2). Notable was the L44H mutation, which generated a CW lock (i.e., exclusively tumbling) phenotype in both CheRB+ and CheRB− cells (Figure 2). Substitution of HR2 in the connector (V33 and I88 in HAMP1 and HAMP2, respectively) tended to increase the CW bias of ATC receptors. Compared to H1, H1 V33G had increased CW bias in CheRB+ cells and was also CW locked in CheRB− cells. H1-2 I88G displayed a slight CW bias in CheRB− cells, which differed from the CCW locked bias of H1-2. Overall, we established a set of HAMP domain mutants with defined phenotypes for structural and biochemical characterization. In general, HAMP1 substitutions that favored CCW output in Tsr could not be overexpressed as soluble proteins when produced in the Aer2 HAMP1 domain, whereas those that produced CW output were generally well tolerated. This suggests that CCW-biasing, but not CW, substitutions disrupt the native HAMP1-like conformation.

### The DExG Motif Rescues Signal Input at the Membrane

**Figure 3:**
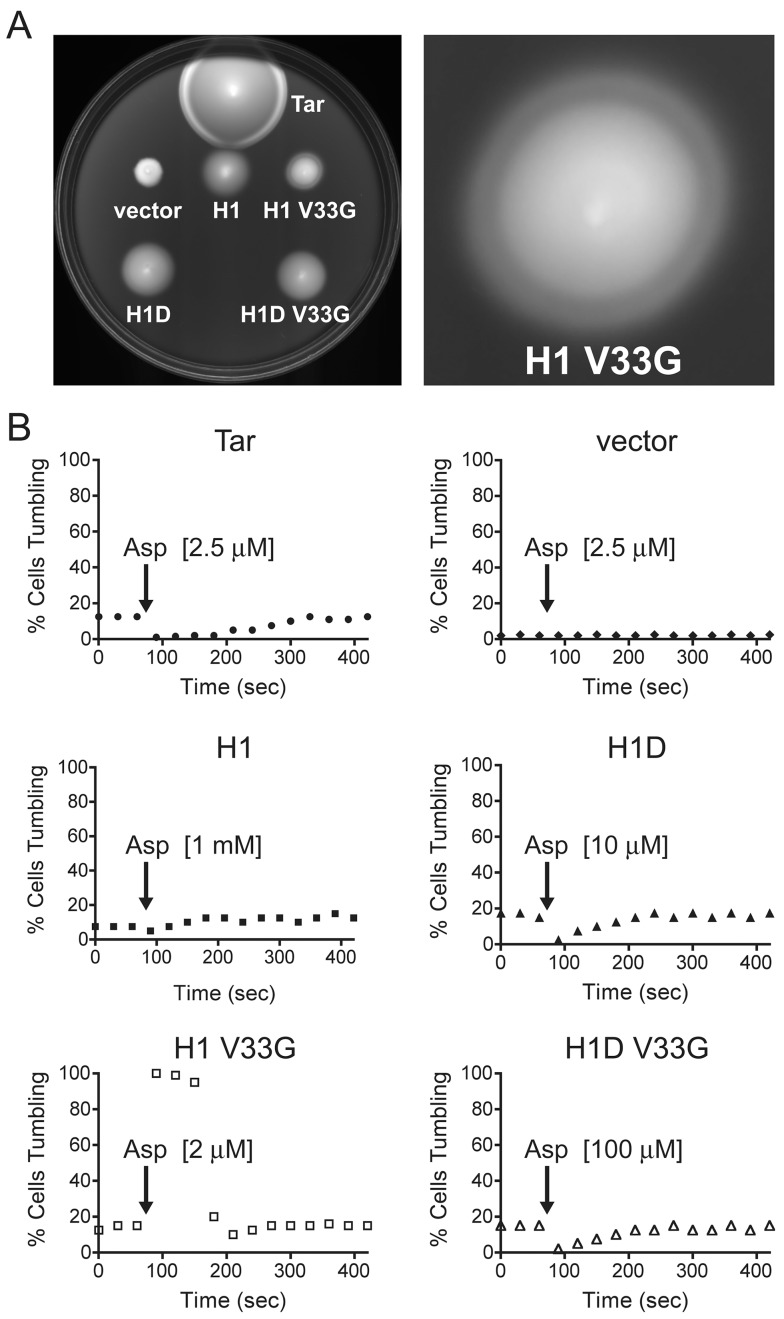
H1D and H1 V33G receptors both respond to attractant, but with normal and inverse responses, respectively. (A) Swim assays of ATC receptors on tryptone agar plates. Colonies with functional chemoreceptors generate a characteristic ring near the leading edge of an expanding colony as cells consume Asp and swim towards higher Asp concentrations. H1 V33G generates an inverted ring, in comparison to Tar, which suggests an inverted CCW-to-CW response to Asp. (B) Temporal assays of transmembrane receptors showing response and adaptation kinetics. CheRB+ cells expressing various receptors were allowed to reach adaptation equilibrium before Asp was added. Tumbling frequencies alter if receptors are capable of receiving and relaying signal input from TM2 to the output KCM. Tar responds in the normal direction, switching from 12.5% to 1% CW bias. After ∼300 s, the adaptation system restores Tar CW bias to 12.5%. H1D has a normal Asp response, switching from 17.5% to 2.5% CW bias. H1 V33G displays an inverted response, switching from 16% to 100% CW bias upon Asp addition. A lower concentration of Asp is representative of increased receptor sensitivity.

To test whether ATCs were capable of receiving and transducing signal input, we carried out temporal assays to monitor flagellar responses to the attractant aspartate (Asp). H1 represented the most promising candidate, as it contained a single HAMP domain and was capable of CheA activation. However, H1 did not switch tumbling bias in response to Asp (Figure 3).

We reasoned this could be due to the lack of two motifs often found in membrane-associated HAMP domains, but not present in the Aer2 HAMPs: (1) a DExG motif at the connector-AS2 junction and (2) a Pro residue at the beginning of AS1 conserved in many HAMP domains, including those of MCPs. Introducing DELG into H1, to produce H1D, generated a functional chemoreceptor with a clear CW-to-CCW switch in response to Asp (Figure 3). In contrast, addition of the proline residue in AS1, to generate H1P, led to an unresponsive CW locked receptor. Combining the two motifs, into H1DP, crippled the previous gain of function. The H1D response was not as robust as that of Tar and required higher Asp concentrations to produce similar kinetics. The H1D mutant introduces an extra residue in AS2 because of a missing residue in this region of native HAMP1. As a control, and to test whether only the highly conserved Glu residue was required for function, we generated H1E, which adds a single Glu residue in the same position (Figure S2). However, H1E failed to respond to Asp.

The DExG motif was introduced into all of the wild-type (WT) ATC receptors to test for functional reconstitution. Unlike H1, the DELG mutation did not affect the signaling bias or the ability of other ATC receptors to respond to Asp. A functional Asp inhibitory response requires the ability to activate CheA. Thus, it was unsurprising that most ATCs remained nonresponsive. Somewhat surprisingly, H1D-2, which has the DExG motif added to HAMP1 in the context of H1-2 and can activate CheA, did not give an attractant response.

We assessed the effects of the DELG mutation on HAMP1 stability in the context of Aer2 1–172 (HAMP1-2/3). WT Aer2 1–172 unfolded in a single step, with a melting temperature (T_M_) of 53°C. In contrast, the H1D protein had two consecutive unfolding steps (Figure S3). At 39°C approximately two-thirds of helical structure was lost, whereas at 65°C the remaining one-third of helical content was lost. These results suggest that the DELG motif decouples HAMP1 from HAMP2/3, rendering HAMP1 with a T_M_ of 65°C and HAMP2/3 with a much lower T_M_ of 39°C. This interpretation derives from the consideration that cooperative unfolding of two-thirds of the helical content implies structural coupling of two adjacent HAMP domains, which are likely to be HAMP2 and HAMP3 as they share a much larger interface than HAMP1 and HAMP2, which are separated by a short linker. Decoupling between HAMP1 and HAMP2/3 is consistent with the lack of Asp response in the H1D-2 receptor, which may not be able to relay a conformational signal through the H1-2 junction. Our attempts to define the molecular basis of these effects were unsuccessful. The H1D protein failed to crystallize, and aggregation of the cysteine-engineered H1D-H1C protein complicated electron spin resonance (ESR) spin-labeling measurements (see below). However, given that H1D imparts signal input to HAMP1, we speculate that these effects may derive from an increased physical connection between the DExG motif and the upstream transmembrane helices.

### V33G Mutation produces a Hyper-Inverted Response to Aspartate

We investigated the ability of other mutations to induce Asp responses. Strikingly, swim assays of H1 V33G displayed a novel phenotype with an inverse ring (Figure 3). Ring formation was validated by addition of Asp at the leading edge of expanding colonies, which caused ring flattening in both Tar and H1 V33G (Figure S4). This odd ring pattern on plates suggested that H1 V33G exhibits an inverted response to Asp. Temporal assays confirmed an inverse Asp response by H1 V33G, in that Asp caused a drastic switch from 16% to 100% CW bias (Figure 3). Notably, H1 V33G had high Asp sensitivity, displaying adaptation kinetics at concentrations similar to those of Tar. The H1D V33G variant, which combines the DELG and V33G substitutions, behaved similarly to H1D, although with significantly decreased sensitivity.

### Crystal Structure of L44H and V33G Mutants Supports HAMP1 as the CW Signaling State

Based on our mutational analysis we aimed to determine the structure of CW locked variants to verify the CW signaling state as a HAMP1-like conformation. Crystals of L44H and V33G proteins were obtained in the context of Aer2 1–172 using conditions similar to those of the native protein [7]. V33G crystallized in the same space group as WT, but L44H produced a different crystal lattice. Complete datasets were collected to 1.9 Å resolution for L44H and to 2.9 Å resolution for V33G, and the structures were determined by molecular replacement.

**Figure 4:**
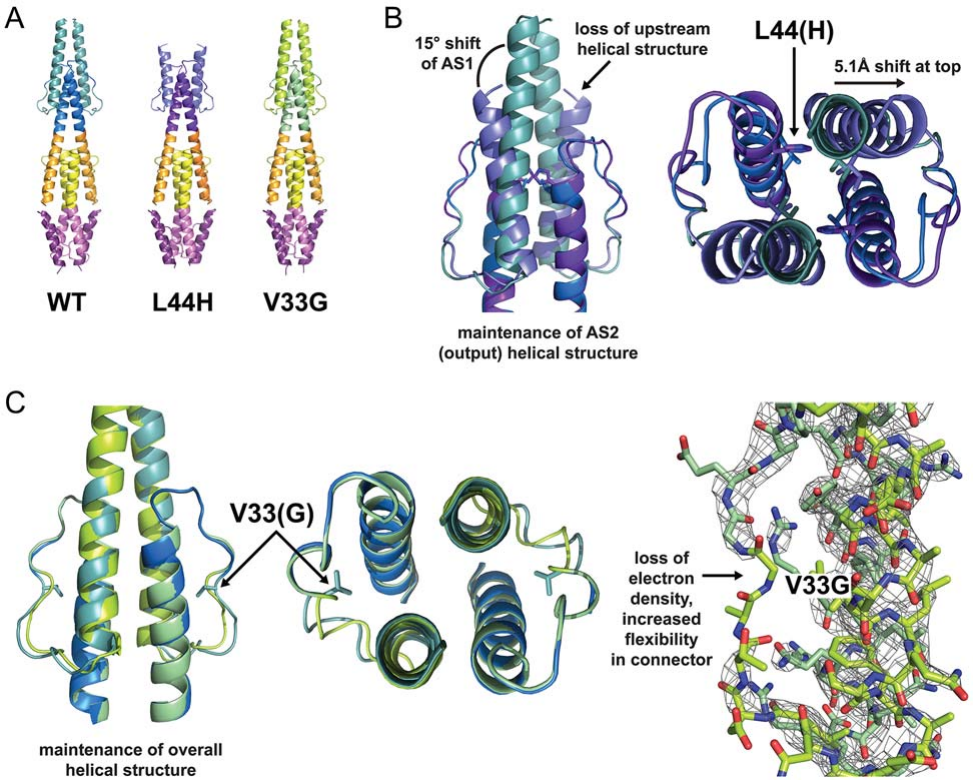
Structure of L44H and V33G mutants. (A) Crystal structure of Aer2 1–172 proteins, with HAMP1 colored blue (WT), purple (L44H), and green (V33G). (B) Superposition of WT HAMP1 and CW locked L44H HAMP1 mutant. The L44H side chain redirects from the bundle core towards AS1, causing a 15° tilt of AS1 away from AS2 and a 5 Å shift at the top of AS1, which disrupts the upstream helical coiled coil. The positions of the AS2 output helices are identical to WT, which confirms that a HAMP1 structure generates CW output. (C) Superposition of HAMP1 in WT and in the inverted signaling V33G mutant. Removal of HR2 in V33G does not affect the helical position of AS1 or AS2, suggesting that HR2 is dispensable to generate a HAMP1 conformer. 2Fo-Fc electron density maps of V33G (contoured at 2 σ) lack density in the surrounding HR2 connector region, suggesting increased flexibility of this region. Increased flexibility of HR2 due to Gly substitution would destabilize a HAMP2 conformer and thereby favor CW output.

The L44H mutation modified the HAMP1 domain structure while leaving the poly-HAMP2/3 domains largely unchanged (Figure 4). The His44 side chain redirected from the bundle core towards AS1. This caused a tilt in the AS1 helix and a 5 Å shift at the top of AS1, resulting in a loss of secondary structure at the AS1 N-terminus. Despite these adjustments in AS1, the AS2 output helices superimposed with those of the native HAMP1 structure. In other words, the mutation did not alter the position of the HAMP1 AS2 helices, which must transmit the CW downstream signal.

The V33G mutation locally destabilized the connector around HR2 and increased mobility in this region of the protein, as evidenced by decreased electron density of the connector in the region of T31–V33 (Figure 4). These changes had no effect on the helical positions of AS1 and AS2 compared to the WT structure, which is consistent with a HAMP1 conformer generating CW output. However, the increased flexibility of the connector and loss of the V33 side chain for packing into the bundle core should affect the ability of HR2 to stabilize the HAMP2 structure, and thus we expected this substitution to disfavor conversion to a HAMP2-like conformer. In Aer2 1–172, HAMP1 and HAMP3 tolerated side chain removal at HR2 but HAMP2 did not (Table S2).

### Soluble ATC Receptors Are Active in Cells and Allow Direct Structure Function Correlations

In addition to full-length transmembrane chimeras, we constructed and assessed the activity of soluble chimeras that had the HAMP1 and HAMP2 domains fused to the Tar KCM (Figure 1). These soluble chimeras, H1s and H1-2s, produced even more distinct phenotypes than their full-length analogs in *E. coli* (Figure 2). Tar KCM produced slight CW bias in CheRB+ cells, but nearly no CW behavior in CheRB− cells. Despite a substantially lower expression level than Tar KCM, H1s generated CW locked behavior in both CheRB+ and CheRB− cells (Figure 2). In contrast, H1-2s was CW biased in CheRB+ cells, but CCW locked in CheRB− cells. These data reinforce the notion that HAMP1 induces a KCM conformation that gives a kinase-on state, and HAMP2 produces a kinase-off state. A striking result is found with H1-2s I88G. This mutation, which would be predicted to destabilize HAMP2, switched the H1-2s phenotype from CCW lock to CW lock in CheRB− cells (Figure 2). The effect was similar, but somewhat muted, in the context of the compensating adaptation system. Importantly, the advantage of the soluble chimeras over their transmembrane counterparts is that their conformational properties can be directly probed in solution with spin-labeling techniques.

### Conformational Properties of Soluble HAMP Domains Fused to Tar

**Figure 5:**
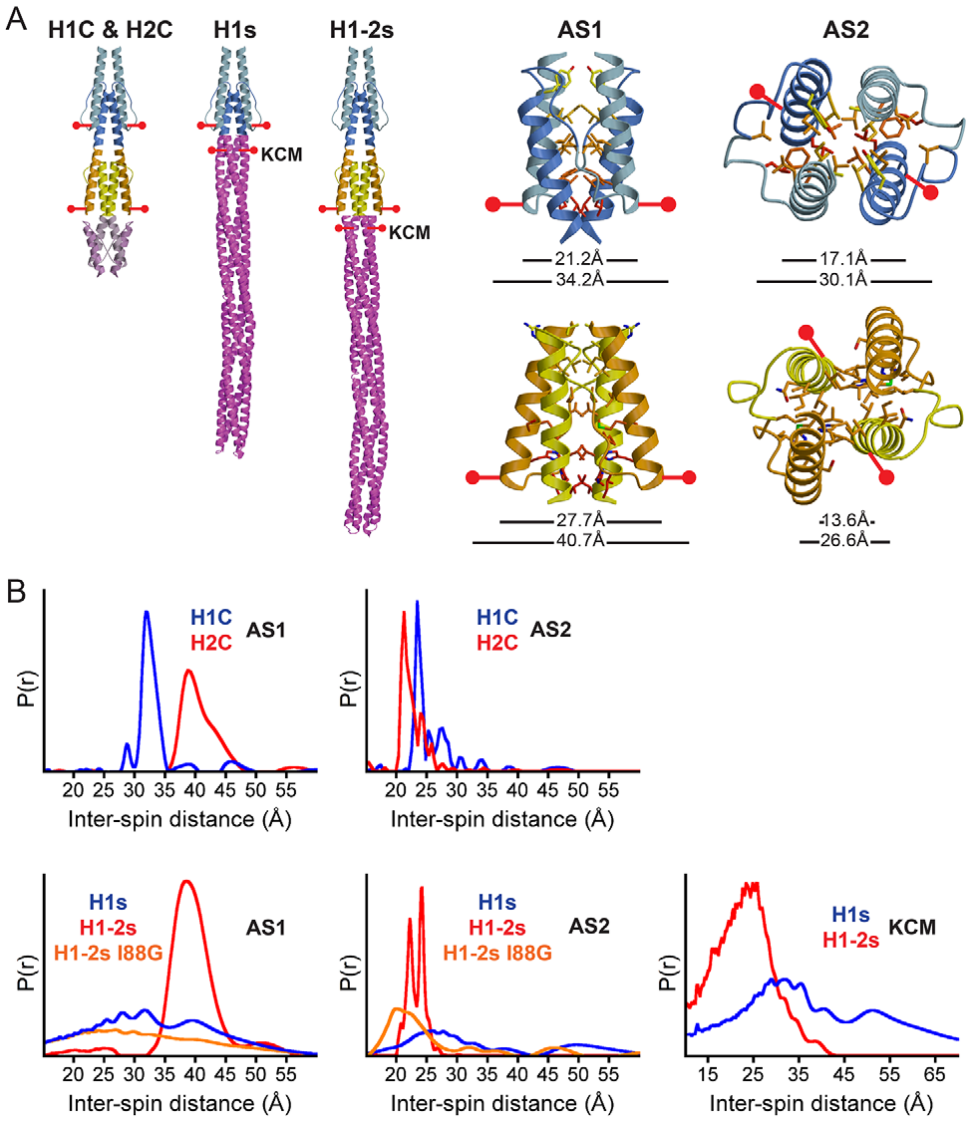
Conformational properties of soluble receptors assessed by PDS. (A) Schematic of spin-label sites in Aer2 1–172 (H1C and H2C) and soluble ATCs (H1s and H1-2s). Sites were chosen in AS1 and AS2 to maximize the distance separation expected to distinguish HAMP1 and HAMP2 in the crystal structure. Cα–Cα distances from the crystal structure are shown (top distance). MTSSL spin labels can add up to 13 Å (bottom distance). (B) Inter-spin distances measured by PDS of spin-labeled proteins. Pair-wise distance distributions (P[r]) of control samples (H1C and H2C) matched well with the differences in the crystal structure (Table 1). Attachment to the Tar KCM (H1s) results in a more dynamic HAMP1 conformer, with broad distance distributions, which is indicative of conformation exchange between HAMP1 and HAMP2. HAMP2, in H1-2s, remains relatively static, with narrow distance peaks that are nearly identical to those of H2C. The H1-2s I88G HR2 mutant switches the conformational properties of HAMP2 towards a dynamic HAMP1 state, consistent with the CW locked phenotype in vivo. The two HAMP conformers have opposite effects downstream. HAMP2, which forms a two-helix coiled coil at the end of AS2, maintains similar distances across the junction in the KCM. HAMP1, which forms a four-helix coiled coil, maintains a longer, broader distance distribution. This suggests the structure of the AS2 helices is propagated downstream into the KCM helical bundle. Inter-spin distance distributions are tabulated in Table 1.

To directly correlate HAMP domain structure with in vivo signaling activity, we measured inter-subunit distance restraints on our soluble variants by site-specific spin labeling and pulsed dipolar ESR spectroscopy (PDS). Nitroxyl spin labels were attached to engineered Cys residues at three positions: (1) the C-terminal end of the AS1 helices, D26C and A81C; (2) the C-terminal end of the AS2 helices, R53C and A109C; and (3) in the KCM bundle directly across from the HAMP junction, E270C (Figure 5). The reporter site in AS1 was chosen because of the large 6.5 Å difference in distance expected between the two conformations, and the reported functional tolerance of this site to mutation in Tsr [6]. The AS2 and KCM sites were chosen to report directly on the conformational changes immediately prior to and following the HAMP/KCM junction. The difference in inter-subunit separation at the AS1 site reflects the change in helix rotation and lateral translation that distinguish HAMP1 and HAMP2 [7]. This change in distance cannot be achieved by rotation of the helices alone. The AS2 site should produce a distinguishable 3.5 Å difference between the separations at the C-terminus for the two conformers and thus report on the signal being relayed to the coupled output domain.

As a control, we first conducted distance measurements of AS1 spin-labeled Aer2 1–172 (referred to as H1C AS1 and H2C AS1) to verify the separations expected by the crystal structure (Figure 5; Table 1). PDS distances of 32.4 Å for H1C AS1 and 39.7 Å for H2C AS1 matched well with the crystal structure separations, given that when combined, the two MTSSL spin labels can add up to 13 Å to the Cα–Cα separation of labeled residues. The 7 Å difference between H1C and H2C easily distinguished the two conformers, and both pair-wise distance distributions had reasonably narrow shapes (Figure 5). In the case of H1C AS1, the sharp peak is characteristic of a single conformation, but for H2C AS1 the broader line shape indicates some contribution from a more separated state of the labels. The closer proximity of the AS2 helices in HAMP2 than in HAMP1 was well reflected by PDS distances of 23.7 Å for H1C AS2 and 21.5 Å for H2C AS2, with both sites reporting narrow peak shapes (Figure 5).

**Table 1:** Inter-spin distance measurements by PDS.

Protein	AS1	AS2	KCM
H1 crystal	21.2 Å	17.1 Å	—
H2 crystal	27.7 Å	13.6 Å	—
H1C^a^	32.4 Å (2.8)	23.7 Å (1.4)	—
H2C^a^	39.7 Å (5.2)	21.5 Å (2.2)	—
H1s^a^	32.6 Å (24.0)	27.1 Å (12.2)	32.1 (22.0)
H1-2s^a^	42.7 Å (7.2)	22.2 Å (1.3), Å (1.1) 24.1	22.4 (14.1) Å
H1-2s I88G^a^	28.9 Å (29.0)	21.5 Å (7.0)	—

Shown are experimentally determined distances of spin-labeled proteins and Cα–Cα distances from the Aer2 1–172 crystal structure. The values shown in parentheses refer to the width (Å) at half the maximum peak height, and qualify peak broadening and conformational heterogeneity. Small values represent narrow peaks and a homogeneous conformation. Large values represent broad peaks consistent with more heterogeneous populations.

a Attachment of the MTSSL spin labels can add up to 13 Å to the Cα–Cα separation, or equivalently 6.5 Å each.

Next we monitored HAMP domain conformations within the soluble Tar KCM fusion receptors H1s and H1-2s (Figure 5; Table 1). The distance distribution of H1-2s AS1 remained centered around 39 Å, but appeared tighter and more symmetric than that of H2C AS1. In contrast, HAMP1 became more conformationally distributed when attached to the Tar KCM than in the context of Aer2 1–172. The H1s AS1 pair-wise distance distribution remained centered on 32 Å but became much broader, with a width at half the maximum peak height of 24.0 Å (Table 1) and two peaks at 32 Å and 39 Å (Figure 5). Thus, the conformation of H1-2s is consistent with a near exclusive HAMP2 conformation, whereas H1s has a broadly distributed conformation centered on a HAMP1-like state but likely also containing contributions from a HAMP2-like state. These structural states correlate well with the opposing CW and CCW locked phenotypes of H1s and H1-2s. Note that the ESR experiment did not measure dynamics directly, but a broad distribution can be reasonably interpreted as a molecule that dynamically exchanges among an ensemble of conformations.

The spin-label sites on AS2 and KCM in H1s and H1-2s produced an interesting similarity in distance and dynamics relative to AS1. H1s AS2 gave a broad distribution centered at 27.1 Å for the inter-subunit distance, but a sharp bimodal distribution centered at 22.2 Å and 24.1 Å for H1-2s AS2 (Figure 5; Table 1). These differences in separation are consistent with the HAMP1 and HAMP2 structures in Aer2 1–172, where, in the case of the latter, the AS2 helices come tightly together to form an effective two-helix coiled coil. The relative separation and dynamics across the KCM junction were maintained, with H1-2s KCM sustaining a sharper distance distribution centered at 22.4 Å, and H1s KCM a longer, broader separation centered at 32.1 Å. Overall, the H1s and H1-2s distance distributions are consistent with near continuous four- and two-helix bundles across the H1/KCM junction, respectively, with the KCM helix retaining the dynamic and static features of the attached HAMP.

### Substitution of HR2 Converts HAMP2 to a HAMP1-Like Conformation and Switches Output

Having established the CW signaling state as a HAMP1 conformer, we aimed to determine the conformational changes associated with the I88G mutation, which changes the behavior of H1-2s from CCW to CW locked in vivo. We reasoned the I88G mutation would alter the conformational equilibrium of HAMP2 to favor a HAMP1-like conformer. Using ESR distance measurements, we analyzed the H1-2s I88G structure in solution. As expected, the I88G mutation destabilized the rigid H1-2s conformation to generate a broad distance distribution (width at half the maximum peak height of 29.0 Å) centered at 28.9 Å for H1-2s I88G AS1 (Table 1). This pair-wise distance distribution was nearly identical to that of H1s AS1 and indicative of conformational exchange (Figure 5). The H1-2s I88G AS2 spin-spin distribution was also broad and overlapped with the distributed signal of H1s AS2, but also contained contribution from a short 21.5 Å distance that is most likely due to direct interactions between the spin labels and the bundle. Although the AS2 conformation in H1-2s I88G may not be identical to that in H1s, it is clearly different from that in H1-2s and shares the distributed properties of that in H1s. Thus, removing HR2 in HAMP2 shifted both receptor bias and HAMP structure toward a CW signaling HAMP1-like conformer. Given the conservation of HR2 and its importance structurally and functionally in CCW signaling, it is possible that other HAMP domains access a HAMP2-like conformation in their signaling mechanisms.

## Discussion

**Figure 6:**
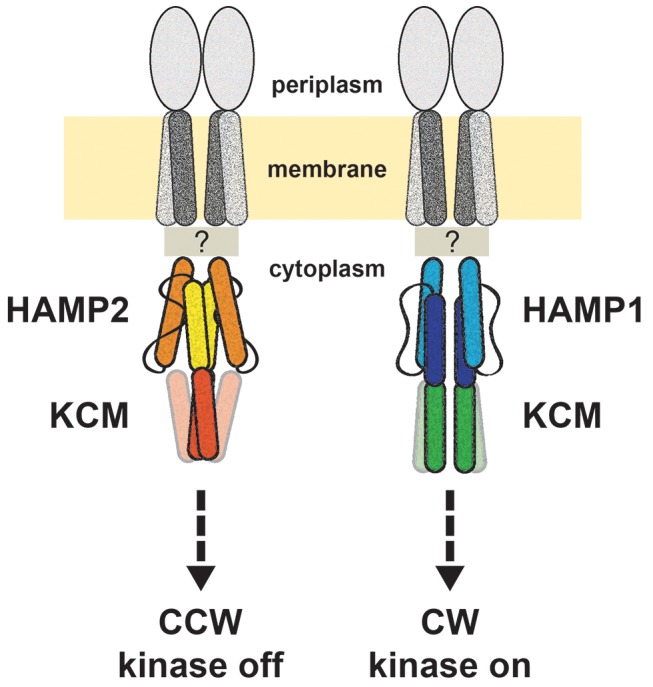
Model for HAMP domain signal relay in bacterial chemoreceptors. The HAMP domains of MCPs exchange between HAMP1 and HAMP2 states to regulate bacterial chemotaxis. The conformation of HAMP2 imparts a two-helix coiled coil across the AS2/KCM junction, which results in CheA kinase inhibition and CCW flagella rotation. A dynamic HAMP1 forms a continuous four-helix coiled coil across the junction to generate kinase activation and CW flagella rotation.

Here we characterize the signaling properties of the Aer2 HAMP domains in chimeric transmembrane receptors and directly correlate structure and dynamics to cellular activities. As previously predicted [7], the HAMP1 and HAMP2 conformations generate CW and CCW signaling biases in bacterial chemoreceptors. Exchange between HAMP conformers is likely sufficient to induce CW biased signaling, whereas a more static HAMP2 conformer generates a CCW signal. Removal of HR2 destabilizes HAMP2, but not HAMP1, altering its structure and signaling bias to resemble those of HAMP1. Physical exchange between HAMP1 and HAMP2 conformers requires a downward motion of AS1 relative to AS2 and is consistent with the downward piston motion of TM2 produced by attractant binding in MCPs [12]. The most straightforward interpretation of our data produces a two-state model in which bacterial chemoreceptors switch primarily between HAMP1- and HAMP2-like states to propagate signals (Figure 6).

The downstream effects of the two HAMP conformers provide important constraints on the output mechanism employed by MCPs. The transition between conformers involves multiple elements including helical translation, rotation, and tilts that are coupled to a rearrangement of the connector/HR2. Most relevant to the activity of downstream effector domains are the changes in position and dynamics of AS2. In HAMP1, the AS2 helices are part of a more standard four-helix bundle, whereas in the relatively distorted HAMP2, the AS2 helices resemble a two-helix coiled coil interaction. Two- and four-helix coiled coils differ with respect to the residues that contribute to the hydrophobic core. In a two-helix coiled coil, the *a* and *d* residues form the core, while in a four-helix coiled coil the *a*, *d*, *e*, and *g* residues can all contribute to the core because of the greater packing contacts among the four helices. HAMP1-to-HAMP2 conversion rotates the AS2 helices in a CCW direction so that a “*g*” position (HAMP1) takes the place of what would otherwise be a “*c*” position (HAMP2) on the core periphery. (This corresponds to about a +55° rotation in Crick angle at the AS2 termini; however, the rotation in HAMP2 is also associated with substantial translation and tilting of the helices.) The HAMP2 AS2 conformation is then in line with the heptad pattern of hydrophobic residues entering the KCM. In the KCM, the *c* positions in-phase with HAMP2 tend to be hydrophilic and would thus disfavor placement as an out-of-phase *g* position. This is consistent with the proposed “stutter compensation” output mechanism based on helical discontinuities at AS2-output helix junctions [7],[20],[23]. The PDS data confirm that the helix separations across the junction follow those of AS2 in the two HAMP conformers, with position 270 appearing more two-helix-like and conformationally rigid when HAMP2 is attached, and more four-helix-like and broader when HAMP1 is attached. Given that the known structures of KCMs are consistently four-helix coiled coils, a switch to a distorted two-helix state would indeed destabilize the four-helix structure. Thus, these structural transitions appear consistent with the yin-yang and biphasic stability models for MCP signal transduction [16],[20],[24], where increased stability in HAMP decreases stability in the KCM that follows.

Note that the KCM of Tar alone causes some CW output, but the kinase-on state is greatly enhanced when the HAMP1 domain is fused to Tar KCM. This stabilization cannot be explained by enhanced dimerization of the KCM because fusing the HAMP1 and HAMP2 domains, which stabilizes the dimer to an even greater extent (as judged by PDS), produces an opposite effect of exclusive CCW output. Furthermore, the expression level of H1s is substantially less than that of the Tar KCM domain itself, yet CW bias is higher; hence the CW lock does not derive from there being high levels of the KCM, which is known to activate CheA [25]. We conclude that HAMP1 exerts some conformational preference on the KCM that activates CheA, even in the absence of the transmembrane and ligand binding regions of Tar.

### Consolidation of HAMP Signaling Models

Exchange between HAMP1 and HAMP2 conformers is also consistent with the biphasic HAMP signaling model [16]. In this case, a HAMP1 conformer would be assigned to the native kinase-on (CW) state. Notably, the PDS distributions of HAMP1 are broader than those of HAMP2, which supports a more dynamic on state predicted by the biphasic model. A HAMP2-like conformer would be assigned to the attractant-mimicking CCW(A) signaling state, given the importance of HR2 to the off state and its role in stabilizing the HAMP2 structure. PDS distributions of HAMP2 were narrower and more conformationally homogeneous than those of HAMP1, which indicates a more stable domain structure and thereby agrees with the increased stability indicated for the CCW(A) state in the biphasic model. The biphasic model also predicts a second CCW(B) state where the HAMP domain is destabilized relative to the CW state. These states are largely found for mutations that are likely to disrupt the HAMP hydrophobic core but leave key hydrophobic residues at the C-terminal end of AS2 intact. Similar types of substitutions introduced into the Aer2 HAMP domains produced proteins that were not well expressed and hence difficult to study, which is consistent with highly destabilized domains. Nonetheless, CCW(B) lesions in Tsr do not completely unfold the HAMP domains because the mutant proteins are still able to exert a kinase-off conformation on the KCM. These results, taken with the structural data presented here, suggest that a key property of any CCW state may be the formation of a tight two-helix bundle at the C-terminal end of AS2. This may be achieved by a range of conformations in the upper HAMP that include those that resemble HAMP2, as well as those that disrupt the upper domain yet still allow close association of the AS2 helices.

Stability may be a difficult parameter to assign to specific HAMP variants, as its formal definition involves the free energy difference between defined states. As all HAMP domains are dynamic to some degree, an ensemble of conformational states is likely involved in their function. With regards to direct measurements of stability, as defined by cooperative helical unfolding, all Aer2 HAMP mutations were destabilizing irrespective of their shift in signaling bias (Figure S5; Table S2). Nonetheless, the HAMP domains of CW output receptors were indeed more dynamic, populating both HAMP1 and HAMP2 conformers. The conformational broadening of HAMP1 observed on fusion to the KCM suggests that an out-of-phase attachment of HAMP to the MCP KCM, which maintains the four-helix structure across the junction, bestows the dynamic properties of the KCM coiled coil onto HAMP. The structure of HAMP2 remains relatively unaffected on fusion to the KCM, but in this case the KCM appears to adopt HAMP2-like properties. Thus, the HAMP domains of MCPs most likely oscillate between two states: a conformationally homogenous CCW state that closely resembles HAMP2 and a more conformationally heterogeneous CW state, whose mean atomic positions resemble HAMP1. In line with the reasoning of Parkinson and Falke and colleagues [16],[20],[24],[26], HAMP1 appears to stabilize the nascent on state inherent to the Tar KCM, whereas the more stable HAMP2 enforces a distorted four-helix bundle across the interface. Notably, the average conformations of the HAMP states and their dynamical properties change together; our data show that an activating HAMP conformation is more dynamic, which does not necessarily mean that any increase in HAMP dynamics is activating.

Studies of Af1503 HAMP fusions to dimerization histidine phosphorylation domains in the context of Taz, a chimera between the Tsr sensing domain and cytoplasmic regions of the sensor kinase EnvZ, found that mutations of key packing residues in the Af1503 HAMP alter the position of AS2 [9]. In particular, a substitution in the bundle core (A291F) that causes a CCW rotation of AS2 (+20° in Crick angle) is more readily able to undergo deactivation by attractant (Ser). Overall, the differences between the variant Af1503 HAMP structures characterized here are more modest than the differences between HAMP1 and HAMP2, and perhaps consistent with this, structural changes are not propagated far across the junction to the dimerization histidine phosphorylation domains. It is difficult to make direct comparison of the activity effects of Tar and Taz, as the signaling modules are quite different; nonetheless, a rotational reorientation of AS2 is a common feature of structures that perturb output in both systems.

### Implications for Other HAMP Systems

Conversion between HAMP1 and HAMP2 states may also apply to other transmembrane receptors. For example, the transmembrane helices of NpHtrII are known to undergo a CW rotation upon light stimulation of the NpSRII-NpHtrII complex [13]. This motion is consistent with the helical rotation of AS1 required to convert between HAMP1 and HAMP2. In addition, the NpHtrII HAMP domain was reported to undergo dynamic oscillation at the C-terminal end of AS1 [27]. HAMP1 and HAMP2 conformational exchange could account for the dynamics of NpHtrII.

Recently Wang et al. [28] reported ESR and labeling measurements using a more stable nanodisc-solubilized NpHtrII HAMP1-2 construct. Contrary to the previous report [27], they did not observe the unstable, dynamic HAMP state that had been seen in different salt concentrations. Upon light stimulation, they did observe alternating helical motions in the two NpHtrII HAMP domains, corresponding to conformational changes consistent with exchange between HAMP1 and HAMP2 conformers. This supports the idea that other HAMP domains may oscillate between HAMP1 and HAMP2 to change output states. Furthermore, the data indicated that signal transduction through tandem HAMP domains involves alternating switching in conformer states [28], as proposed from the Aer2 structures [7].

Although our model can be applied beyond the scope of MCPs, it is not clear if all ∼26,000 identified HAMP domains utilize the same conformational signaling mechanism. Previous studies involving chimera transmembrane receptors suggest some HAMP domains share a conserved mechanism [29]. We report here that with minor modification the soluble HAMP domains of Aer2 can function within transmembrane chemoreceptors and respond to ligand in both normal and inverse directions. However, as we have seen with HAMP1, attachment to up- and downstream domains may influence the conformation and/or dynamic properties of HAMP domains. Thus, although there is a significant body of evidence that HAMP domains are interchangeable modules sharing a conserved mechanism, it is possible these findings derive from a plastic property of HAMP domains that allows them to be molded in various ways by each input and output domain to which they are attached. For example, the large, flexible MCP KCMs may bestow dynamic properties upon MCP HAMP domains that are not found in sensor kinase HAMP domains.

### The DExG Motif Distinguishes Membrane-Associated and Poly-HAMP Domains

The region that distinguishes canonical, membrane-associated, and divergent poly-HAMP domains is the connector-AS2 junction [17]. Canonical HAMPs contain the DExG motif, while divergent HAMPs conserve a single glycine [7]. Our finding that addition of the DExG motif reconstitutes transmembrane function into the divergent and soluble HAMP1 suggests that these two HAMP subtypes are distinguished mainly by their mode of signal input. Canonical HAMP domains require the DExG motif to couple to upstream transmembrane signals. In contrast, divergent HAMP domains require the conserved glycine to pack closely in a poly-HAMP chain. Currently, the role the DExG motif plays is unclear. In the Af1503 HAMP the conserved Glu of this motif hydrogen-bonds with the N-terminus of AS1 (Figure S6) and thereby may couple conformational signals coming from the transmembrane helices into the connector. Alternatively, the motif may tune the conformational equilibrium of the on and off states to make the off state more accessible to perturbations induced by ligand binding.

### HAMP Domain Mutational Effects

The L44H structure provides new insight into the structural consequences of HAMP domain residue substitutions that perturb function. In the L44H structure, the tilt of AS1 drives the helices apart, disrupting upstream helical packing and resulting in a loss of observed secondary structure at the N-terminus of AS1. In the context of a transmembrane MCP, if this helix disruption were maintained, it would decouple TM2 from AS1. Because the L44H variant is strongly CW biasing, and the H1 conformation generates CW output, we assume that the structure seen at the C-terminal domain in the crystal is maintained in the Tar fusions. However, within a transmembrane MCP, it is also possible that similar mutations maintain the TM2/AS1 junction and that the strain induced by the substitution disrupts, rather, the connectivity of the AS2/KCM junction. This idea offers the possibility that the phenotypes of some MCP mutants may derive from disruptions at the up- or downstream HAMP domain junctions and subsequent decoupling of signal input and output. Thus, it is perhaps not surprising that different types of residue substitutions at the same position can produce very different phenotypes, as seen in Tsr [16],[20]. Likewise, for similar reasons, the effects of several substitutions at different sites may not necessarily be additive. Such complex behavior results when the H1D substitution is present along with the L44H, V33G, or I88G substitutions (Figure 2).

### Inverted Signaling of H1 V33G: Potential Mechanisms and Application

The mechanism underlying H1 V33G inverted signaling is not completely understood; however, it is clear that a branched hydrophobic residue at HR2 is important to achieve the HAMP2 conformation and CCW signaling state. Thus, it is perhaps reasonable that upon attractant binding, the V33G variant is unable to switch to a HAMP2 conformation. Unable to obtain the HAMP2 state, upstream perturbation causes the equilibrium to shift toward HAMP1. Stabilization of a HAMP1 state by V33G is evident by the effect of this mutation in the CheRB− background, where it produces an exclusively CW state. Nonetheless, introduction of the DExG motif overcomes the V33G lesion and restores a normal CCW response to attractant. Thus, the DExG motif must stabilize a CCW state despite the absence of HR2. Overall, the effects of these lesions underscore the fine balance between the CW and CCW conformational states that HAMP domains assume and the cooperative contributions of many residues to their relative stabilities and transitions.

Finally, the H1 V33G HAMP domain may provide a useful tool for engineering receptor-driven processes in bacteria. Substitution of H1D and H1 V33G into chimeric chemoreceptors should produce opposite chemotactic responses to the same ligand. This strategy could be applied to direct genetically modified bacteria towards or away from specific chemicals. This may be especially advantageous in remediation efforts for taxing bacteria towards chemicals that are normally repellants.

## Materials and Methods

### Bacterial Strains

ATC expression and behavioral assays mainly utilized two *E. coli* strains: BT3388 (*tar*, *tsr*, *trg*, *tap*, *aer*) [30] and a Δ*cheRB* strain UU2610 (*tar*, *tsr*, *trg*, *tap*, *aer*, *cheR*, *cheB*) (a gift from J. S. Parkinson), both of which lack all native chemoreceptors. For isogenic comparison, the *E. coli* strain UU2612 (*tar*, *tsr*, *trg*, *tap*, *aer*) was used (J. S. Parkinson).

### Cloning and Mutation

*E. coli* Tar was cloned from genomic DNA into Litmus 28i with 5′ XbaI-NdeI and 3′ HindIII-XhoI restriction sites. Silent mutations were utilized to remove an internal NdeI site in Tar and to introduce BamHI and PmlI sites near the 5′ and 3′ boundaries of the Tar HAMP domain. To generate ATCs, Aer2 HAMP fragments were cloned into the engineered BamHI and PmlI sites of Tar/Litmus 28i. Final ATCs replaced the Tar HAMP domain (214–262) with Aer2 HAMP domains: H1 (8–56), H2 (63–112), H3 (109–156), H1-2 (8–56), H1-23 (8–156), and H23 (63–156). Full-length ATC receptors were transferred using NdeI and HindIII sites to the vector pKG116, which contained a salicylate inducible promoter. Soluble ATCs for in vivo studies were generated by ligating an Aer2 PCR fragment with a NdeI and PmlI digested Tar/pKG116 vector. For ESR studies, soluble ATCs were transferred to pET28 using NdeI and HindIII sites. All HAMP domain mutations were introduced using either the QuikChange strategy or overlap extension. A full list of sequences and primers is reported in Text S1. The correct sequence for all clones was confirmed by direct nucleotide sequencing.

### Quantification of Cell Tumbling Frequencies

Qualitative experiments were first carried out using standard swim assays in tryptone semisoft agar supplemented with 12.5 µg/ml chloramphenicol and 0.5 or 1 µM sodium salicylate. Plates were incubated at 30°C for 15–19 h. Aspartate rings were verified by placing 2 µl of 0.5 M aspartate on top of the semisoft agar, ∼2 mm in front of the leading colony edge, and incubating plates for a further 5 h. Direct measurements of cell tumbling frequencies were carried out using temporal assays. *E. coli* cells harboring ATC plasmids were grown in tryptone broth, induced for 1 h with 2 µM sodium salicylate, washed and resuspended in KEP buffer (10 mM potassium phosphate, 0.1 mM EDTA [pH 7.0]), and then visualized by dark-field microscopy. Cells reached adaptation equilibrium after 5 min, after which cell tumbling frequencies were measured. The ability of ATC receptors to respond to aspartate was tested using temporal assays combined with monitoring of changes in tumbling frequency after the addition of various aspartate concentrations.

### Expression Levels of ATC Receptors

Expression levels of proteins in BT3388 cells were analyzed by Western blotting after induction with 2 µM sodium salicylate, using antisera against the highly conserved region of Tsr (common to all chemoreceptors) (a gift from J. S. Parkinson). Bands were visualized on Western blots and quantified on a BioSpectrum digital imager (UVP).

### Circular Dichroism Spectroscopy

Circular dichroism experiments on HAMP domain mutants were carried out using a AVIV Biomedical (model 202-01) spectropolarimeter. The protein sample (∼0.5 mg/ml, in 10 mM sodium phosphate buffer [pH 7.5]) was heated 1°C per min and allowed to reach equilibrium for 2 min. After that, the degree of ellipticity was measured, averaged over 1 min, and plotted versus temperature.

### Crystallization and Data Collection

Aer2 1–172 mutant proteins were purified as previously described for the WT protein [7] with the exception of induction temperature, which was reduced to 18°C. Crystals of Aer2 1–172 V33G protein were obtained in conditions and space group identical to those described previously for the WT protein [7]. Aer2 1–172 L44H protein (40 mg/ml) crystallized in a different space group (P3_2_12). L44H crystals were grown by vapor diffusion, mixing 1.5 µl of protein with 1.5 µl of well solution, against a reservoir containing 1.5–1.7 M MgSO_4_ and 0.1 M Tris (pH 8.5) for 6–10 h at room temperature. Diffraction data were collected at the Cornell High Energy Synchrotron Source A1 beamline on an ADSC Quantum 210 CCD detector. Data were processed with HKL2000 [31].

### Structure Determination and Refinement

V33G and L44H structures were determined by molecular replacement using Phenix AutoMR [32]. The structures of V33G and L44H were built using XFIT [33] and COOT [34], respectively, and structure refinement was carried out using CNS [35] and Phenix [32], respectively, amidst cycles of manual model building, minimization, B-factor refinement, and solvent molecule placement to produce the final models (V33G, R-factor = 23.5%, *R*_free_ = 28.0%; L44H R-factor = 20.8%, *R*_free_ = 25.9%) (Table S3).

### Preparation of Spin-Labeled Proteins

All soluble ATC receptors were overexpressed in *E. coli* BL21 (DE3) cells at room temperature for 6–18 h using IPTG. Proteins were purified using a gravity Ni-column and size-exclusion chromatography on a Superdex 200 Hi-Load 26/60 column. Aer2 and Tar lack any native cysteine residues. Site-directed mutagenesis introduced cysteine residues for spin labeling in HAMP1 and HAMP2 at AS1 (D26 and A81) and AS2 (E53 and A107). A cysteine residue at E270 in Tar KCM was introduced two helix turns from the AS2/KCM junction, which starts at Tar D263. Spin labeling was accomplished as previously described [36] by incubating protein and MTSSL spin label with gentle mixing for 4 h at room temperature (H1C and H2C) or overnight at 4°C (H1s and H1-2s). Excess spin label was removed by buffer exchange using a desalting column. ESR measurements were conducted within 24 h of spin labeling, or protein was flash-frozen and thawed within 1 wk to ensure sample quality.

### PDS Measurements

PDS measurements were conducted at the Advanced Electron Resonance Technology facility as previously described [36],[37]. Double electron electron resonance experiments were carried out at 17.35 GHz on a home-built 2D-FT ESR spectrometer, with either 16-ns or 32-ns pump pulses [38]. Protein concentrations were in the range of 25–50 µM. Dipolar evolution times were typically about 2.5 microseconds. The baseline was approximated by a linear polynomial in most cases. Subsequently, distance distributions were calculated by Tikhonov regularization [39] and further refined by a maximum entropy regularization method [40].

### Accession Numbers

The atomic coordinates and structure factors of the L44H and V33G crystal structures have been deposited in the Protein Data Bank (http://www.rcsb.org/pdb/home/home.do) under accession codes 4I3M and 4I44.

## Supporting Information

Figure S1 **Swim assays of ATCs.** Swim assays could distinguish between CheA inhibiting (CCW), CheA activating (CW), and functional receptors. H1 and H1-2, which have HAMP1 and HAMP2 attached to the KCM domain of Tar, exhibit similar downstream signals in adaptation-proficient cells (CheRB+) but opposite signals in CheRB− cells.
(TIF)

Figure S2 **HAMP domain alignment.** HAMP domain alignment highlighting the location of HR2, the CW locked L44H mutation, the DExG motif, the conserved glycine in divergent HAMPs, and ESR spin-labeling sites. The H1D mutant introduces an extra residue in AS2 of HAMP1; however, H1E, which also adds an extra residue, failed to switch in response to aspartate.
(TIF)

Figure S3 **The DELG mutation decouples HAMP1 from HAMP2/3.** Circular dichroism thermal melting curves of Aer2 1–172 WT and H1D proteins. WT protein unfolds in a single step and has a melting temperature of 53**°**C. H1D protein unfolds in two steps, one at 39**°**C and another at 65**°**C, which account for 2/3 and 1/3 of secondary structure, respectively. This suggests that the H1D mutation stabilizes HAMP1 and additionally decouples HAMP1 from HAMP2/3.
(TIF)

Figure S4 **Verification of aspartate rings by ring flattening.** Aspartate rings were verified by a flattening of the expanding ring after placing 2 µl of 0.5 M Asp on top of the semisoft agar, ∼2 mm in front of the leading colony edge, and incubating plates for a further 5 h. Arrows highlight the flattened ring, which confirms the normal and inverse Asp responses of Tar and H1 V33G.
(TIF)

Figure S5 **Melting curves of HAMP1 mutants.** Circular dichroism thermal melting curves of Aer2 1–172 proteins. All mutations, with the exception of H1D, destabilized Aer2, resulting in a lower melting temperature. Overall, there was no correlation between stability and signaling bias.
(TIF)

Figure S6 **The Glu in the DExG motif hydrogen-bonds to AS1 in the Af1503 structure.** Structure of Af1503 (Protein Data Bank code 2ASW) highlighting 2.7 Å hydrogen bond between E311 and carbonyl (T281) in AS1 [5].
(TIF)

Table S1 **Tumbling biases of ATC receptors.** Tumbling biases were determined by temporal assays.
(DOCX)

Table S2 **Tumbling biases of ATC mutant receptors.** Tumbling biases were determined by temporal assays. Melting temperatures of HAMP mutants that could be successfully overexpressed in the context of Aer2 1–172 are shown. Some mutations resulted in insoluble protein upon overexpression. The extensive mutational library of Tsr mutants was used to select mutations and is shown for comparison.
(DOCX)

Table S3 **Data collection and refinement statistics.**
(DOCX)

Text S1 **Nucleotide sequences of ATC receptors and a list of primers used in this study.**
(DOCX)

## Abbreviations

ATC: Aer2–Tar chimera
CCW: counterclockwise
CW: clockwise
ESR: electron spin resonance
KCM: kinase control module
MCP: methyl-accepting chemotaxis protein
NMR: nuclear magnetic resonance
PDS: pulsed dipolar electron spin resonance spectroscopy
WT: wild-type
