# Supplementary figures and images for: HAMP Domain Conformers That Propagate Opposite Signals in Bacterial Chemoreceptors
Source: PLoS Biol. 2013 Feb 12;11(2):e1001479. doi: 10.1371/journal.pbio.1001479 (PMC3570549; doi:10.1371/journal.pbio.1001479)

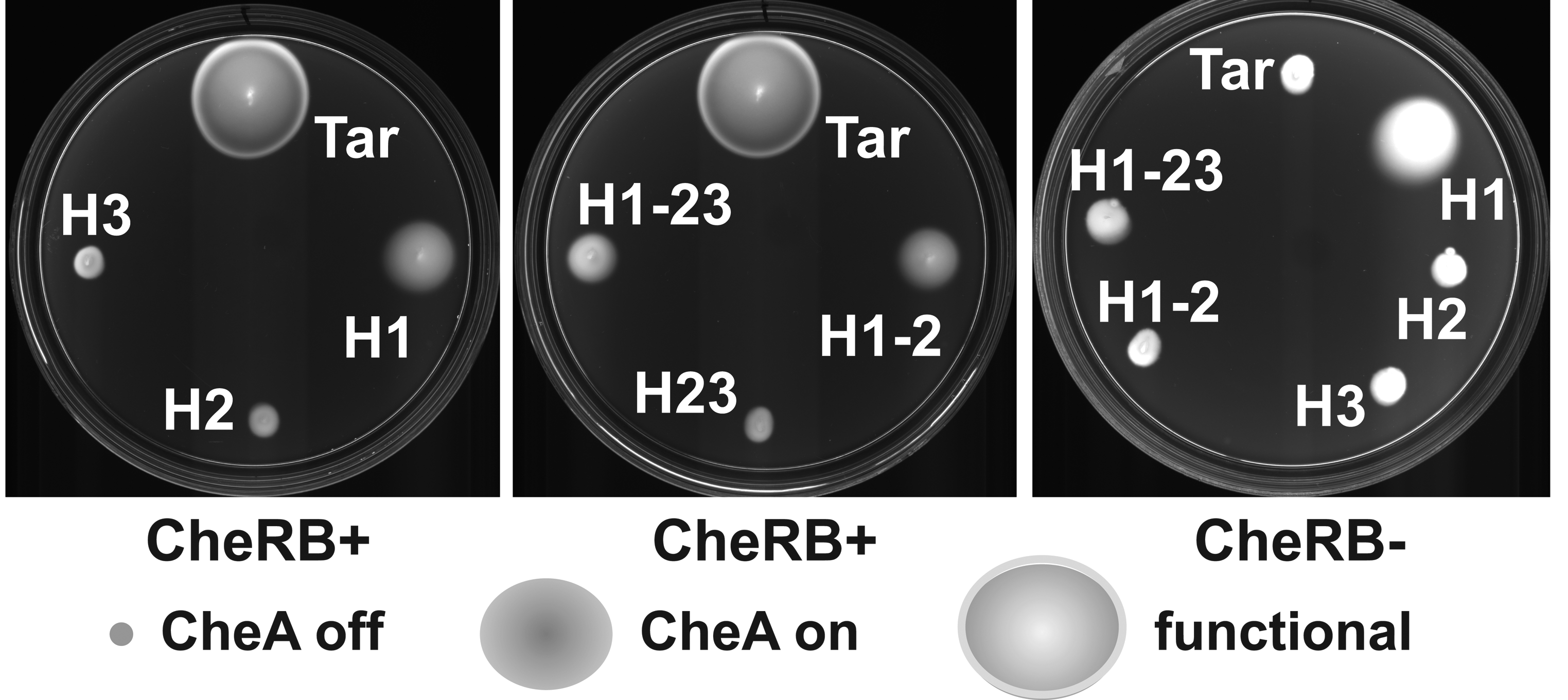

Supplement: Figure S1 — Swim assays of ATCs. Swim assays could distinguish between CheA inhibiting (CCW), CheA activating (CW), and functional receptors. H1 and H1-2, which have HAMP1 and HAMP2 attached to the KCM domain of Tar, exhibit similar downstream signals in adaptation-proficient cells (CheRB+) but opposite signals in CheRB− cells. (TIF) [file pbio.1001479.s001.tif]

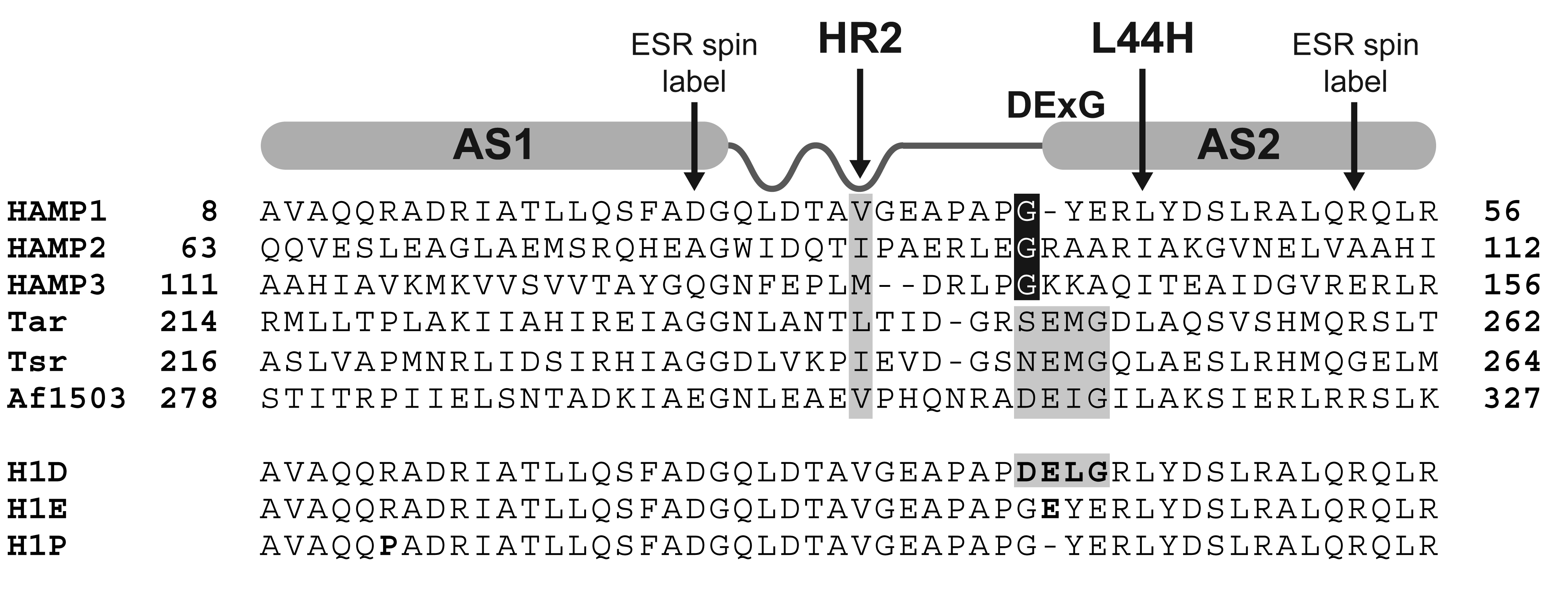

Supplement: Figure S2 — HAMP domain alignment. HAMP domain alignment highlighting the location of HR2, the CW locked L44H mutation, the DExG motif, the conserved glycine in divergent HAMPs, and ESR spin-labeling sites. The H1D mutant introduces an extra residue in AS2 of HAMP1; however, H1E, which also adds an extra residue, failed to switch in response to aspartate. (TIF) [file pbio.1001479.s002.tif]

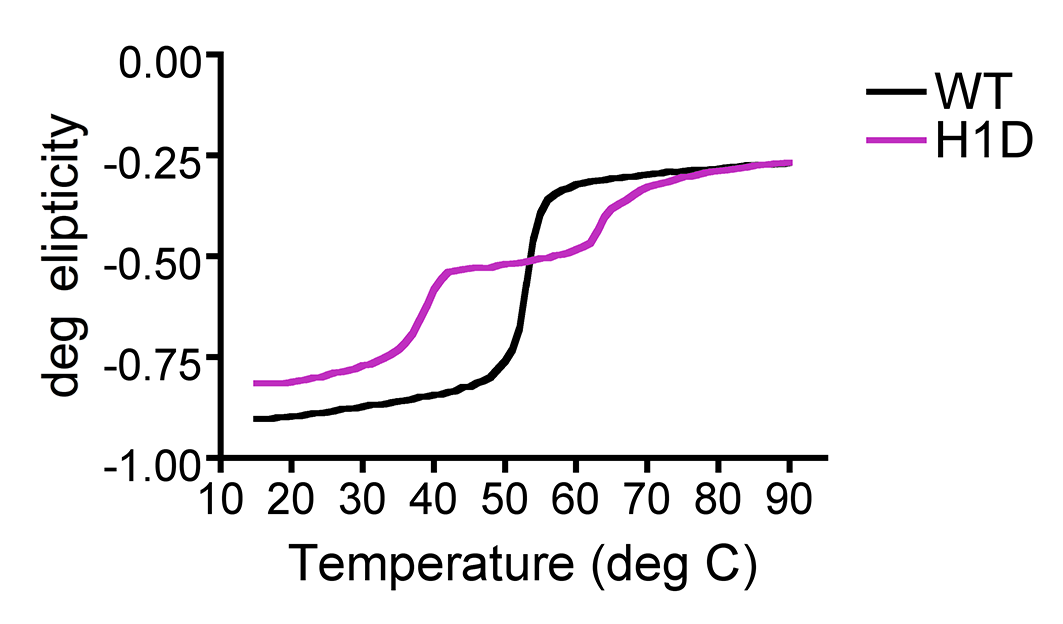

Supplement: Figure S3 — The DELG mutation decouples HAMP1 from HAMP2/3. Circular dichroism thermal melting curves of Aer2 1–172 WT and H1D proteins. WT protein unfolds in a single step and has a melting temperature of 53°C. H1D protein unfolds in two steps, one at 39°C and another at 65°C, which account for 2/3 and 1/3 of secondary structure, respectively. This suggests that the H1D mutation stabilizes HAMP1 and additionally decouples HAMP1 from HAMP2/3. (TIF) [file pbio.1001479.s003.tif]

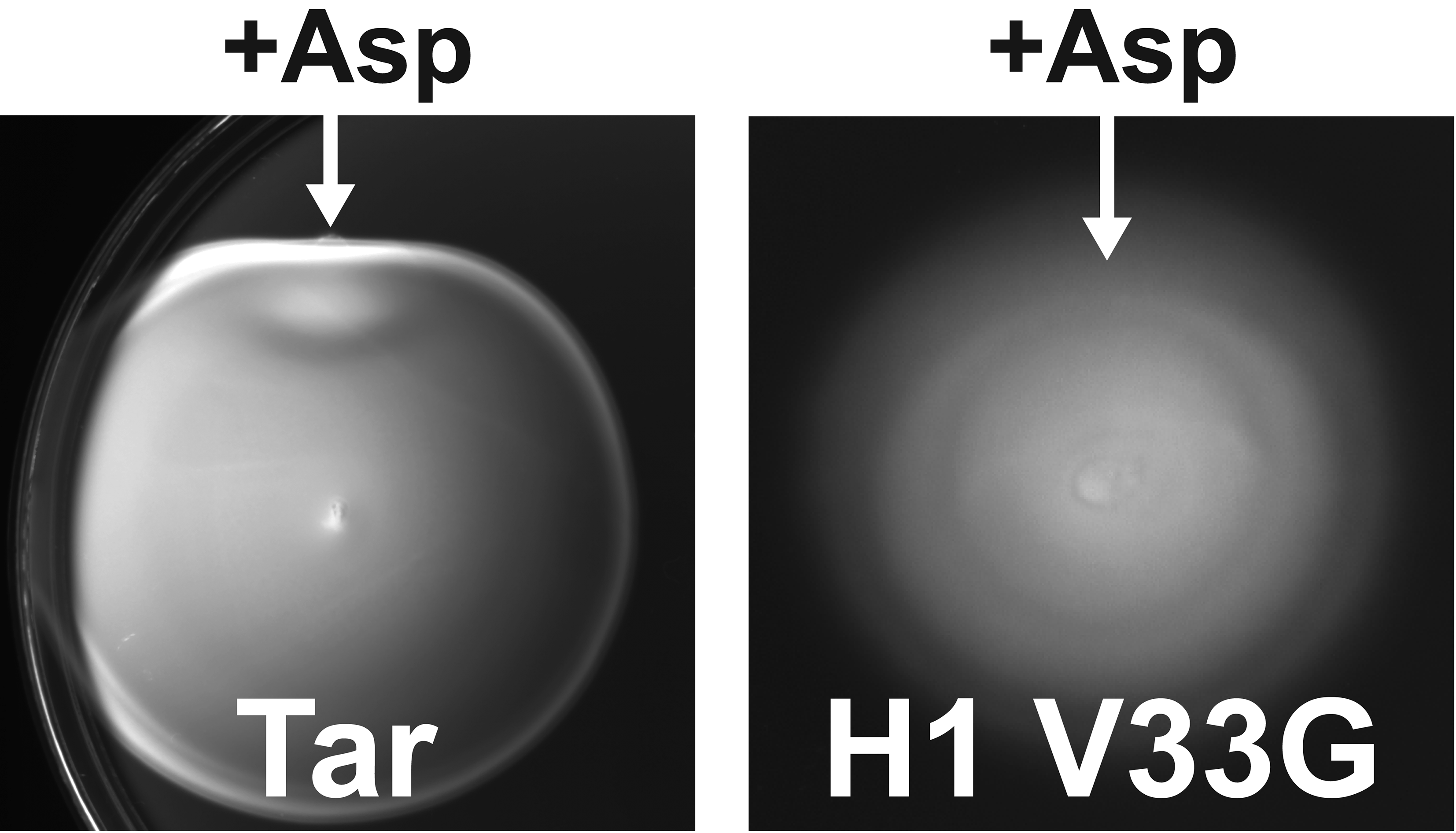

Supplement: Figure S4 — Verification of aspartate rings by ring flattening. Aspartate rings were verified by a flattening of the expanding ring after placing 2 µl of 0.5 M Asp on top of the semisoft agar, ∼2 mm in front of the leading colony edge, and incubating plates for a further 5 h. Arrows highlight the flattened ring, which confirms the normal and inverse Asp responses of Tar and H1 V33G. (TIF) [file pbio.1001479.s004.tif]

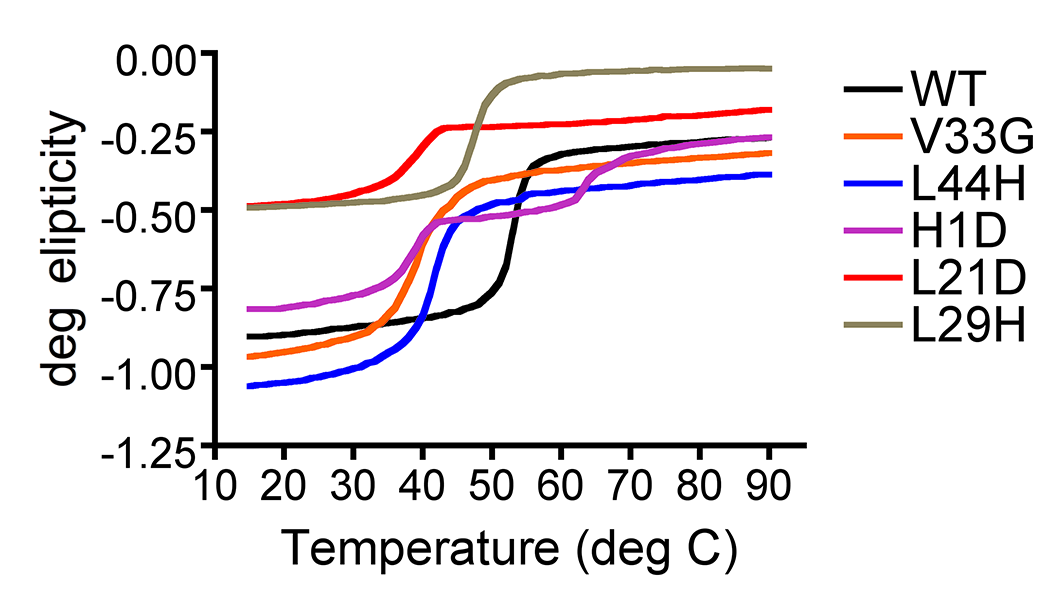

Supplement: Figure S5 — Melting curves of HAMP1 mutants. Circular dichroism thermal melting curves of Aer2 1–172 proteins. All mutations, with the exception of H1D, destabilized Aer2, resulting in a lower melting temperature. Overall, there was no correlation between stability and signaling bias. (TIF) [file pbio.1001479.s005.tif]

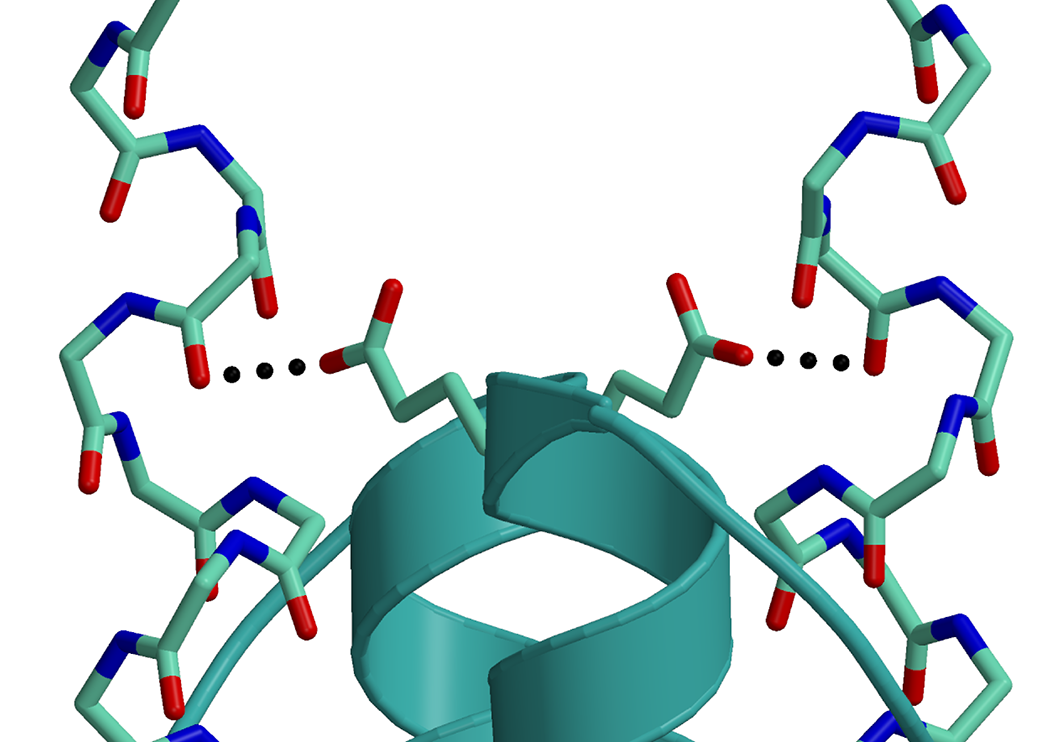

Supplement: Figure S6 — The Glu in the DExG motif hydrogen-bonds to AS1 in the Af1503 structure. Structure of Af1503 (Protein Data Bank code 2ASW) highlighting 2.7 Å hydrogen bond between E311 and carbonyl (T281) in AS1 [5]. (TIF) [file pbio.1001479.s006.tif]
