# Supplementary material for: HAMP Domain Conformers That Propagate Opposite Signals in Bacterial Chemoreceptors
Source: PLoS Biol. 2013 Feb 12;11(2):e1001479. doi: 10.1371/journal.pbio.1001479 (PMC3570549; doi:10.1371/journal.pbio.1001479)
Supplement: Table S1 — Tumbling biases of ATC receptors. Tumbling biases were determined by temporal assays. (DOCX) [file pbio.1001479.s007.docx]

**Table S1. Tumbling biases of ATC receptors.** Tumbling biases were determined by temporal assays.

| **ATC** | **CheRB+**  **(BT3388)** | **CheRB-**  **(UU2610)** |
| --- | --- | --- |
| Vector | CCW bias | CCW bias |
| Tar | CW bias | CW lock |
| H1 | Slight CW bias | CW lock |
| H2 | CCW bias | CCW bias |
| H3 | Slight CW bias | Slight CW bias |
| H1-2 | Slight CW bias | CCW bias |
| H23 | CCW bias | Not tested |
| H1-23 | CCW bias | CCW bias |
