# Supplementary material for: HAMP Domain Conformers That Propagate Opposite Signals in Bacterial Chemoreceptors
Source: PLoS Biol. 2013 Feb 12;11(2):e1001479. doi: 10.1371/journal.pbio.1001479 (PMC3570549; doi:10.1371/journal.pbio.1001479)
Supplement: Table S2 — Tumbling biases of ATC mutant receptors. Tumbling biases were determined by temporal assays. Melting temperatures of HAMP mutants that could be successfully overexpressed in the context of Aer2 1–172 are shown. Some mutations resulted in insoluble protein upon overexpression. The extensive mutational library of Tsr mutants was used to select mutations and is shown for comparison. (DOCX) [file pbio.1001479.s008.docx]

**Table S2. Tumbling biases of ATC mutant receptors.** Tumbling biases were determined by temporal assays. Melting temperatures of HAMP mutants, that could be successfully overexpressed in the context of Aer2 1-172, are shown. Some mutations resulted in insoluble protein upon overexpression. The extensive mutational library of Tsr mutants was used to select mutations and is shown for comparison.

| **HAMP Protein** | **Melting Temp (°C)** | **CheRB+ (BT3388)** | **CheRB- (UU2610)** | **Tsr Phenotype** | |
| --- | --- | --- | --- | --- | --- |
| Tar | - | CW bias | CW lock | - |  |
| H1 | 53 | Slight CW bias | CW lock | - |  |
| L21D | 39 | CCW bias | CCW bias | CCW^(^[^1^](#_ENREF_1)^)^ |  |
| L29H | 47 | CCW bias | Slight CW bias | CCW lock^(^[^2^](#_ENREF_2)^)^ |  |
| V33G | 39 | CW bias | CW lock | CW lock^(^[^2^](#_ENREF_2)^)^ |  |
| L44H | 43 | Strong CW bias | CW lock | CCW^(^[^1^](#_ENREF_1)^)^ |  |
| H1D | 39, 65 | CW bias | Strong CW bias | - |  |
| L44N | Insoluble | Strong CW bias | Not tested | CCW^(^[^1^](#_ENREF_1)^)^ |  |
| L48E | Insoluble | CCW bias | Not tested | CCW^(^[^1^](#_ENREF_1)^)^ |  |
| L48G | Insoluble | CCW bias | Not tested | CCW^(^[^1^](#_ENREF_1)^)^ |  |
| L48Y | Insoluble | CW lock | CW lock | CCW^(^[^1^](#_ENREF_1)^)^ |  |
| H2 | - | CCW bias | CCW bias | - |  |
| H2-I88G | Insoluble | CCW bias | CCW bias | - |  |
| H1-2 | - | Slight CW bias | CCW bias | - |  |
| H1-2 I88G | Insoluble | CCW bias | Slight CW bias | - |  |

1. Zhou Q, Ames P, & Parkinson JS (2011) Biphasic control logic of HAMP domain signalling in the Escherichia coli serine chemoreceptor. *Molecular Microbiology* 80(3):596-611.

2. Ames P, Zhou Q, & Parkinson JS (2008) Mutational analysis of the connector segment in the HAMP domain of Tsr, the Escherichia coli serine chemoreceptor. *Journal of Bacteriology* 190(20):6676-6685.
