# Supplementary material for: HAMP Domain Conformers That Propagate Opposite Signals in Bacterial Chemoreceptors
Source: PLoS Biol. 2013 Feb 12;11(2):e1001479. doi: 10.1371/journal.pbio.1001479 (PMC3570549; doi:10.1371/journal.pbio.1001479)
Supplement: Table S3 — Data collection and refinement statistics. (DOCX) [file pbio.1001479.s009.docx]

**Table S3. Data collection and refinement statistics.**

| **Data Collection** |  |  | | |
| --- | --- | --- | --- | --- |
|  | L44H | | | V33G |
| Wavelength (Å) | 0.97918 | | | 0.97857 |
| Space group | P3_2_12 | | | P4_3_2_1_2 |
| Cell parameters (Å) | a = b = 61.1, c = 81.4 | | | a = b = 113.4, c = 65.0 |
| Resolution (Å) | 50-1.95 (1.98-1.95) | | | 50-2.88 (2.93-2.88) |
| No. of reflections | 138172 | | | 96286 |
| No. of unique reflections | 12812 | | | 10197 |
| Completeness (%) | 99.7 (100.0) | | | 99.4 (100.0) |
| R_sym_ ^a^ | 0.074 (0.366) | | | 0.040 (0.349) |
| I/σ(I) | 30.6 (6.8) | | | 50.2 (8.4) |
|  | | |  | |
| **Refinement statistics** |  | | |  |
| Resolution range (Å) | 50-1.95 Å (1.98-1.95) | | | 50.0-2.88 Å (2.93-2.88) |
| R factor, % | 20.8 (21.2) | | | 23.5 (32.9) |
| R_free_, % | 25.9 (27.0) | | | 28.0 (35.0) |
| Atoms (protein, solvent) | 1149, 178 | | | 1229, 14 |
| Mean B-values (Å^2^) |  | | |  |
| Protein | 34.7 | | | 85.8 |
| Solvent | 51.3 | | | 65.0 |
| R.m.s. deviations |  | | |  |
| Bond lengths (Å) | 0.004 Å | | | 0.007 Å |
| Bond angles (deg) | 0.98 deg | | | 1.21 deg |
| Missing residues | 1-6, 157-172 | | | 157-172 |
| *Highest resolution shell is shown in parenthesis | | | | |
