# Supplementary material for: HAMP Domain Conformers That Propagate Opposite Signals in Bacterial Chemoreceptors
Source: PLoS Biol. 2013 Feb 12;11(2):e1001479. doi: 10.1371/journal.pbio.1001479 (PMC3570549; doi:10.1371/journal.pbio.1001479)
Supplement: Text S1 — Nucleotide sequences of ATC receptors and a list of primers used in this study. (DOCX) [file pbio.1001479.s010.docx]

**Text S1.** Nucleotide sequences of ATC receptors and a list of primers used in this study**.**

**SEQUENCES**

**Full-length receptors: Tar, H1, H2, H3, H1-2, H23, and H1-23**

EcTar-NdeIout-BamHIadd-PmlIadd = “Tar”

atgattaaccgtatccgcgtagtcacgctgttggtaatggtgctgggggtattcgcactgttacagcttatttccggcagtctgtttttttcttcccttcaccatagccagaagagctttgtggtttccaatcaattacgggaacagcagggcgagctgacgtcaacctgggatttaatgctgcaaacgcgcattaacctgagtcgttcagcggtacggatgatgatggattcctctaatcaacaaagtaacgccaaagttgaattgctcgatagcgccaggaaaacattggcgcaggcagcgacgcattataaaaaattcaaaagcatggcaccgttacctgaaatggtcgctaccagtcgtaatattgatgaaaaatataaaaactattacacagcgttaactgaactgattgattatctagattatggcaatactggagcttatttcgctcagccaacccagggaatgcaaaatgcaatgggcgaagcgtttgctcagtacgccctcagcagtgaaaaactgtatcgcgatatcgtcactgacaacgcagatgattaccgatttgcccagtggcaactggcggttatcgcgctggtggtggtattgattctgctggtggcgtggtacg**ggatcc**gc*cgtatgttgcttactccgctggcaaaaattattgctcacattcgcgaaatcgccggtggtaacctggcgaataccctgaccattgacgggcgcagtgaaatgggcgacctggcgcagagcgtttcacacatgcaacgctctttgact*gacaccgtcact**cacgtg**cgcgaaggttcagatgccatctatgccggtacccgtgaaattgcggcgggcaacaccgatctttcctcccgtactgaacagcaggcatccgcgctggaagaaactgccgccagcatggagcagctcaccgcgacagtgaagcaaaacgccgataacgcccgccaggcctcgcaactggcgcaaagtgcctccgacaccgcccagcacggcggcaaagtggtggatggcgtagtgaaaacgatgcatgagatcgccgatagttcgaagaaaattgccgacattatcagcgttatcgacggtattgccttccagactaatatcctcgcgctgaatgccgcggttgaagccgcgcgtgcgggtgaacagggccgtggttttgccgtggtggcgggtgaagtgcgtaatcttgccagtcgcagcgcccaggcggcaaaagagatcaaagccctcattgaagactccgtctcacgcgttgataccggttcggtgctggtcgaaagcgccggggaaacaatgaacaatatcgtcaatgctgtcactcgcgtgactgacattatgggcgagattgcatcggcatcggatgaacagagccgtggcatcgatcaagtcgcattggcggtttcggaaatggatcgcgtcacgcaacagaacgcatcgctggtgcaggaatcagctgccgccgccgctgcgctggaagaacaggcgagtcgtttaacgcaagcggtttccgcgttccgtctggcagccagcccactcaccaataaaccgcaaacaccatcccgtcctgccagtgagcaaccaccggcacagccacgactgcgaattgctgaacaagatccaaactgggaaacattttga

H1: (replace italic sequence with) *gcggtggcgcagcaacgcgccgaccggatcgccacgctcctccagtcgttcgccgacggccagctcgacaccgccgtcggcgaggccccggcgcccggctacgagcgtctctacgacagcctgcgggcgctgcagcggcaactgcgc*

H2: (replace italic sequence with) *cagcaggtggaaagcctggaggcggggctggcggagatgagccgccagcacgaagccggctggatcgaccagacgattcccgccgaacgcctggaaggtcgcgcggcgcgcatcgccaagggcgtcaacgaactggtggccgcgcacatc*

H3: (replace italic sequence with) *gccgcgcacatcgcggtgaagatgaaggtggtcagcgtggtcaccgcctacggccagggcaacttcgagccgctgatggaccgcctgccgggcaagaaggcgcagatcaccgaagccatcgacggcgttcgcgaacgcctgcgc*

H1-2: (replace italic sequence with) *gcggtggcgcagcaacgcgccgaccggatcgccacgctcctccagtcgttcgccgacggccagctcgacaccgccgtcggcgaggccccggcgcccggctacgagcgtctctacgacagcctgcgggcgctgcagcggcaactgcgcgaacaacgcgccgaactccagcaggtggaaagcctggaggcggggctggcggagatgagccgccagcacgaagccggctggatcgaccagacgattcccgccgaacgcctggaaggtcgcgcggcgcgcatcgccaagggcgtcaacgaactggtggccgcgcacatc*

H23: (replace italic sequence with) *cagcaggtggaaagcctggaggcggggctggcggagatgagccgccagcacgaagccggctggatcgaccagacgattcccgccgaacgcctggaaggtcgcgcggcgcgcatcgccaagggcgtcaacgaactggtggccgcgcacatcgcggtgaagatgaaggtggtcagcgtggtcaccgcctacggccagggcaacttcgagccgctgatggaccgcctgccgggcaagaaggcgcagatcaccgaagccatcgacggcgttcgcgaacgcctgcgc*

H1-23: (replace italic sequence with) *gcggtggcgcagcaacgcgccgaccggatcgccacgctcctccagtcgttcgccgacggccagctcgacaccgccgtcggcgaggccccggcgcccggctacgagcgtctctacgacagcctgcgggcgctgcagcggcaactgcgcgaacaacgcgccgaactccagcaggtggaaagcctggaggcggggctggcggagatgagccgccagcacgaagccggctggatcgaccagacgattcccgccgaacgcctggaaggtcgcgcggcgcgcatcgccaagggcgtcaacgaactggtggccgcgcacatcgcggtgaagatgaaggtggtcagcgtggtcaccgcctacggccagggcaacttcgagccgctgatggaccgcctgccgggcaagaaggcgcagatcaccgaagccatcgacggcgttcgcgaacgcctgcgc*

**Soluble receptors: Tar SD, H1s, H1-2s, and H1-23s**

Tar SD:

atggacaccgtcactcacgtgcgcgaaggttcagatgccatctatgccggtacccgtgaaattgcggcgggcaacaccgatctttcctcccgtactgaacagcaggcatccgcgctggaagaaactgccgccagcatggagcagctcaccgcgacagtgaagcaaaacgccgataacgcccgccaggcctcgcaactggcgcaaagtgcctccgacaccgcccagcacggcggcaaagtggtggatggcgtagtgaaaacgatgcatgagatcgccgatagttcgaagaaaattgccgacattatcagcgttatcgacggtattgccttccagactaatatcctcgcgctgaatgccgcggttgaagccgcgcgtgcgggtgaacagggccgtggttttgccgtggtggcgggtgaagtgcgtaatcttgccagtcgcagcgcccaggcggcaaaagagatcaaagccctcattgaagactccgtctcacgcgttgataccggttcggtgctggtcgaaagcgccggggaaacaatgaacaatatcgtcaatgctgtcactcgcgtgactgacattatgggcgagattgcatcggcatcggatgaacagagccgtggcatcgatcaagtcgcattggcggtttcggaaatggatcgcgtcacgcaacagaacgcatcgctggtgcaggaatcagctgccgccgccgctgcgctggaagaacaggcgagtcgtttaacgcaagcggtttccgcgttccgtctggcagccagcccactcaccaataaaccgcaaacaccatcccgtcctgccagtgagcaaccaccggcacagccacgactgcgaattgctgaacaagatccaaactgggaaacattttga

H1s:

**atgggtctgttcaatgcacatgcggtggcgcagcaacgcgccgaccggatcgccacgctcctccagtcgttcgccgacgg**

**ccagctcgacaccgccgtcggcgaggccccggcgcccggctacgagcgtctctacgacagcctgcgggcgctgcagcggc**

**aactgcgc**gacaccgtcactcacgtgcgcgaaggttcagatgccatctatgccggtacccgtgaaattgcggcgggcaacaccgatctttcctcccgtactgaacagcaggcatccgcgctggaagaaactgccgccagcatggagcagctcaccgcgacagtgaagcaaaacgccgataacgcccgccaggcctcgcaactggcgcaaagtgcctccgacaccgcccagcacggcggcaaagtggtggatggcgtagtgaaaacgatgcatgagatcgccgatagttcgaagaaaattgccgacattatcagcgttatcgacggtattgccttccagactaatatcctcgcgctgaatgccgcggttgaagccgcgcgtgcgggtgaacagggccgtggttttgccgtggtggcgggtgaagtgcgtaatcttgccagtcgcagcgcccaggcggcaaaagagatcaaagccctcattgaagactccgtctcacgcgttgataccggttcggtgctggtcgaaagcgccggggaaacaatgaacaatatcgtcaatgctgtcactcgcgtgactgacattatgggcgagattgcatcggcatcggatgaacagagccgtggcatcgatcaagtcgcattggcggtttcggaaatggatcgcgtcacgcaacagaacgcatcgctggtgcaggaatcagctgccgccgccgctgcgctggaagaacaggcgagtcgtttaacgcaagcggtttccgcgttccgtctggcagccagcccactcaccaataaaccgcaaacaccatcccgtcctgccagtgagcaaccaccggcacagccacgactgcgaattgctgaacaagatccaaactgggaaacattttga

H1-2s: (replace bold sequence from H1s with)

**atgggtctgttcaatgcacatgcggtggcgcagcaacgcgccgaccggatcgccacgctcctccagtcgttcgccgacggccagctcgacaccgccgtcggcgaggccccggcgcccggctacgagcgtctctacgacagcctgcgggcgctgcagcggcaactgcgcgaacaacgcgccgaactccagcaggtggaaagcctggaggcggggctggcggagatgagccgccagcacgaagccggctggatcgaccagacgattcccgccgaacgcctggaaggtcgcgcggcgcgcatcgccaagggcgtcaacgaactggtggccgcgcacatc**

H1-23s: (replace bold sequence from H1s with)

**atgggtctgttcaatgcacatgcggtggcgcagcaacgcgccgaccggatcgccacgctcctccagtcgttcgccgacggccagctcgacaccgccgtcggcgaggccccggcgcccggctacgagcgtctctacgacagcctgcgggcgctgcagcggcaactgcgcgaacaacgcgccgaactccagcaggtggaaagcctggaggcggggctggcggagatgagccgccagcacgaagccggctggatcgaccagacgattcccgccgaacgcctggaaggtcgcgcggcgcgcatcgccaagggcgtcaacgaactggtggccgcgcacatcgcggtgaagatgaaggtggtcagcgtggtcaccgcctacggccagggcaacttcgagccgctgatggaccgcctgccgggcaagaaggcgcagatcaccgaagccatcgacggcgttcgcgaacgcctgcgc**

**PRIMER LIST**

**Cloning**

EcTar-1-XbaINdeF: gccgactctagacatatgattaaccgtatccgcgtagtc

EcTar-553-HindIIIXhoR: gccgacaagcttctcgagtcaaaatgtttcccagtttggatctt

EcTar-NdeIOut-F: gcgcagagcgtttcacacatgcaacgctctttg

EcTar-NdeIOut-R: caaagagcgttgcatgtgtgaaacgctctgcgc

EcTar-BamMutSite-F: gtggtacgggatccgccgtatgttgcttactccg

EcTar-BamMutSite-R: catacggcggatcccgtaccacgccaccagcaga

EcTar-PmlIMutSite-F: cgtcactcacgtgcgcgaaggttcagatgccatc

EcTar-PmlIMutSite-R: accttcgcgcacgtgagtgacggtgtcagtcaaa

ATC-H1-8-F: gccgacggatccgcgcggtggcgcagcaacgcgcc

ATC-H1-56-R: gccgaccacgtgagtgacggtgtcgcgcagttgccgctgcagcgc

ATC-H2-63-F: gccgacggatccgccagcaggtggaaagcctggag

ATC-H2-112-R: gccgaccacgtgagtgacggtgtcgatgtgcgcggccaccagttc

ATC-H3-109-F: gccgacggatccgcgccgcgcacatcgcggtgaag

ATC-H3-156-R: gccgaccacgtgagtgacggtgtcgcgcaggcgttcgcgaacgcc

ATC-H1s-Nde-F: gacgcccatatgggtctgttcaatgcacat

**Mutagenesis**

H1D-F: gaggccccggcgcccgacgaactagggcgtctctacgacagc

H1D-R: gctgtcgtagagacgccctagttcgtcgggcgccggggcctc

H2D-F: gccgaacgcctggaagatgaactggggcgcatcgccaagggc

H2D-R: gcccttggcgatgcgccccagttcatcttccaggcgttcggc

H3D-F: atggaccgcctgccggacgagctggggcagatcaccgaagcc

H3D-R: ggcttcggtgatctgccccagctcgtccggcaggcggtccat

H1E-F: ccggcgcccggcgagtacgagcgtctctacgacagc

H1E-R: gagacgctcgtactcgccgggcgccggggcctcgcc

H1P-R13P--F: gcggtggcgcagcaacccgccgaccggatcgcc

H1P-R13P-R: gagcgtggcgatccggtcggcgggttgctgcgc

V33G-F: gacggccagctcgacaccgccggcggcgaggcc

V33G-R: gccgggcgccggggcctcgccgccggcggtgtc

V33G-Fb: accgccggaggcgaggccccg

V33G-Rb: ctcgcctccggcggtgtcgagct

I88G-F: gccggctggatcgaccagacgggtcccgccgaa

I88G-R: ccttccaggcgttcggcgggacccgtctggtcg

L29H-F: cagtcgttcgccgacggccagcacgacaccgcc

L29H-R: ggcctcgccgacggcggtgtcgtgctggccgtc

L44H-F: ccggcgcccggctacgagcgtcactacgacagc

L44H-R: cagcgcccgcaggctgtcgtagtgacgctcgta

L44N-F: ccggcgcccggctacgagcgtaactacgacagc

L44N-R: cagcgcccgcaggctgtcgtagttacgctcgta

L48E-F: tacgagcgtctctacgacagctaccgggcgctg

L48E-R: cagttgccgctgcagcgcccgctcgctgtcgta

L48G-F: tacgagcgtctctacgacagcgggcgggcgctg

L48G-R: cagttgccgctgcagcgcccgcccgctgtcgta

L48Y-F: tacgacagctaccgggcgctgcagcggcaactg

L48Y-R: cagttgccgctgcagcgcccggtagctgtcgta

H1s-D26C-F: acgctcctccagtcgttcgcctgcggccagctc

H1s-D26C-R: gacggcggtgtcgagctggccgcaggcgaacga

H1s-R53C-F: cgctgcagtgccaactgcgcgaacaacgc

H1s-R53C-R: cgcagttggcactgcagcgcccgcag

H1-2s-A81C-F: gagatgagccgccagcacgaatgcggctggatc

H1-2s-A81C-R: aatcgtctggtcgatccagccgcattcgtgctg

H1-2s-A81C-(I88G)-R: acccgtctggtcgatccagccgcattcgtgctg

H1-2s-A109C-F: gaactggtgtgcgcgcacatcgcggtgaag

H1-2s-A109C-R: gatgtgcgcgcacaccagttcgttgacgcc
